# Identification of Plasmonic Modes in Parabolic Cylinder Geometry by Quasi-Separation of Variables

**DOI:** 10.1007/s11468-014-9791-3

**Published:** 2014-10-01

**Authors:** Kazuyoshi Kurihara, Akira Otomo, Kazuhiro Yamamoto, Junichi Takahara, Masahiko Tani, Fumiyoshi Kuwashima

**Affiliations:** 1 Department of Physics, Faculty of Education and Regional Studies, University of Fukui, Fukui, 910-8507 Japan; 2 Kobe Advanced ICT Research Center, National Institute of Information and Communications Technology (NICT), Kobe, 651-2492 Japan; 3 Institute for Materials Chemistry and Engineering, Kyushu University, Fukuoka, 816-8580 Japan; 4 Graduate School of Engineering, Osaka University, 1-2 Yamadaoka, Suita, Osaka 565-0871 Japan; 5 Research Center for Development of Far-Infrared Region, University of Fukui, Fukui, 910-8507 Japan; 6 Department of Electrical, Electronics and Computer Engineering, Fukui University of Technology, Fukui, 910-8505 Japan

**Keywords:** Surface plasmon polaritons, Plasmonics, Quasi-separation of variables, Parabolic cylinder, Nanofocusing, Superfocusing

## Abstract

This paper describes the plasmonic modes in the parabolic cylinder geometry as a theoretical complement to the previous paper (J Phys A 42:185401) that considered the modes in the circular paraboloidal geometry. In order to identify the plasmonic modes in the parabolic cylinder geometry, analytic solutions for surface plasmon polaritons are examined by solving the wave equation for the magnetic field in parabolic cylindrical coordinates using quasi-separation of variables in combination with perturbation methods. The examination of the zeroth-order perturbation equations showed that solutions cannot exist for the parabolic metal wedge but can be obtained for the parabolic metal groove as standing wave solutions indicated by the even and odd symmetries.

## Introduction

Metallic tapered waveguides have attracted considerable attention as a possible experimental structure for the waveguides in achieving deep subwavelength confinement not only in optical region [1, 2] but also in terahertz (THz) region [3–5]. Theoretical investigation of the metallic tapered waveguides has been conducted so far in the context of superfocusing beyond the diffraction limit of focused electromagnetic waves [6–8]. Superfocusing in metallic tapered waveguides originates from two remarkable theoretical papers [9, 10] in 1997 regarding the peculiarities of surface plasmon polaritons (SPPs) [11, 12] known as electromagnetic waves propagating along a metal-dielectric interface. The first [9] predicted subwavelength waveguides of SPPs by J. Takahara (one of the present authors) and his colleagues, whereas the second [10] introduced the concept of superfocusing for SPPs at the apexes of metallic tapered waveguides. These papers together provide a complete conceptual description of SPPs in metallic tapered waveguides at both extreme sides. A substantive issue in theoretical physics is to identify the plasmonic modes in metallic tapered waveguides that clarify the intermediate behavior of SPPs propagating from an infinite distance to the apex of tapered waveguides.

Our approach to finding plasmonic modes in metallic tapered waveguides is to solve a wave equation by the quasi-separation of variables (QSOV) [13–15] in combination with the use of perturbation methods, which is based on the idea of smoothly connecting analytic solutions at both extreme sides of metallic tapered waveguides. The QSOV technique was first applied to conical geometry [13] and then to wedge-shaped geometry [14], with unprecedented results of plasmonic modes that enable us to consider superfocusing properties with respect to the taper angle in conical- and wedge-shaped geometries. A remaining important topic in metallic tapered waveguides is to investigate the effect of round tips of tapered waveguides, which is very difficult to consider using conventional analytical methods based on classical separation of variables (CSOV) such as the geometrical-optics (GO) approximation (also called adiabatic approximation) [16–27] and local separation of variables (LSOV) approximation [10, 28–31]. In a previous paper [15], we successfully applied the QSOV technique to a circular paraboloidal geometry, which is one of the simplest tapered geometries with a finite tip radius, allowing us to discover that despite the metallic tapered waveguide, superfocusing does not take place in this geometry. Some of the knowledge is practically useful for analysis of THz experimental results in the circular paraboloidal geometry [32]. To properly understand the effect of round tips of metallic tapered waveguides, more theoretical investigation using the QSOV technique is needed into the tapered geometries with finite tip radii.

In this paper, we describe a theoretical investigation of plasmonic modes in a parabolic cylinder geometry by solving the wave equation for the magnetic field by means of the QSOV in combination with perturbation methods. We observed that plasmonic modes cannot exist for the parabolic metal wedge but can be obtained for the parabolic metal groove as standing waves classified by even and odd symmetries. This result is remarkably similar to that in the circular paraboloidal geometry [15], where we previously observed that the plasmonic modes cannot exist for the metallic solid paraboloid but can be obtained for the metallic hollow paraboloid as standing waves. Importantly, the superfocusing does not take place in the parabolic cylinder geometry as well as in the circular paraboloidal geometry [15]; hence, from a parabolical standpoint, the present paper is interrelated and complementary to the previous paper [15] treating the circular paraboloidal geometry. The new knowledge obtained in this paper is a fundamental building block in plasmonics and is practically useful for explaining numerical simulation through metallic subwavelength structures consisting partly of parabolic cylinder geometry, as described in [33–35].

## Quasi-Separation of Variables Applied to the Wave Equation for a Magnetic Field

**Fig. 1:**
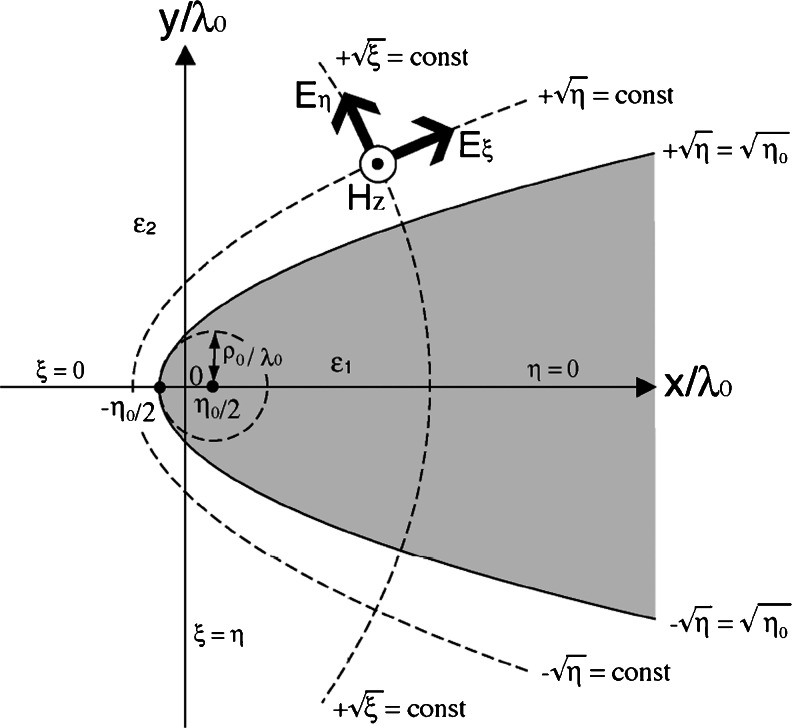
Geometry of a parabolic cylinder structure for the parabolic metal wedge and the parabolic metal groove. *ρ*_0_ is the radius of curvature for the parabolic cylinder structure at the apex. *ε*_1_ and *ε*_2_ are the permittivities inside and outside the parabolic cylinder, respectively. *H*_*z*_ is the magnetic field of SPPs with translational symmetry about the *z*-axis. *E*_*ξ*_ and *E*_*η*_ are the electric fields of the radial and angular components, respectively, induced by the magnetic filed *H*_*z*_. $+\sqrt{\eta }=\sqrt{\eta_0}$ and $-\sqrt{\eta }=\sqrt{\eta_0}$ are the equations for the upper and lower surfaces, respectively, of the parabolic cylinder structure. Geometric dimensions of the *x*- and *y*-axes are normalized by the wavelength in vacuum, *λ*_0_

**Fig. 2:**
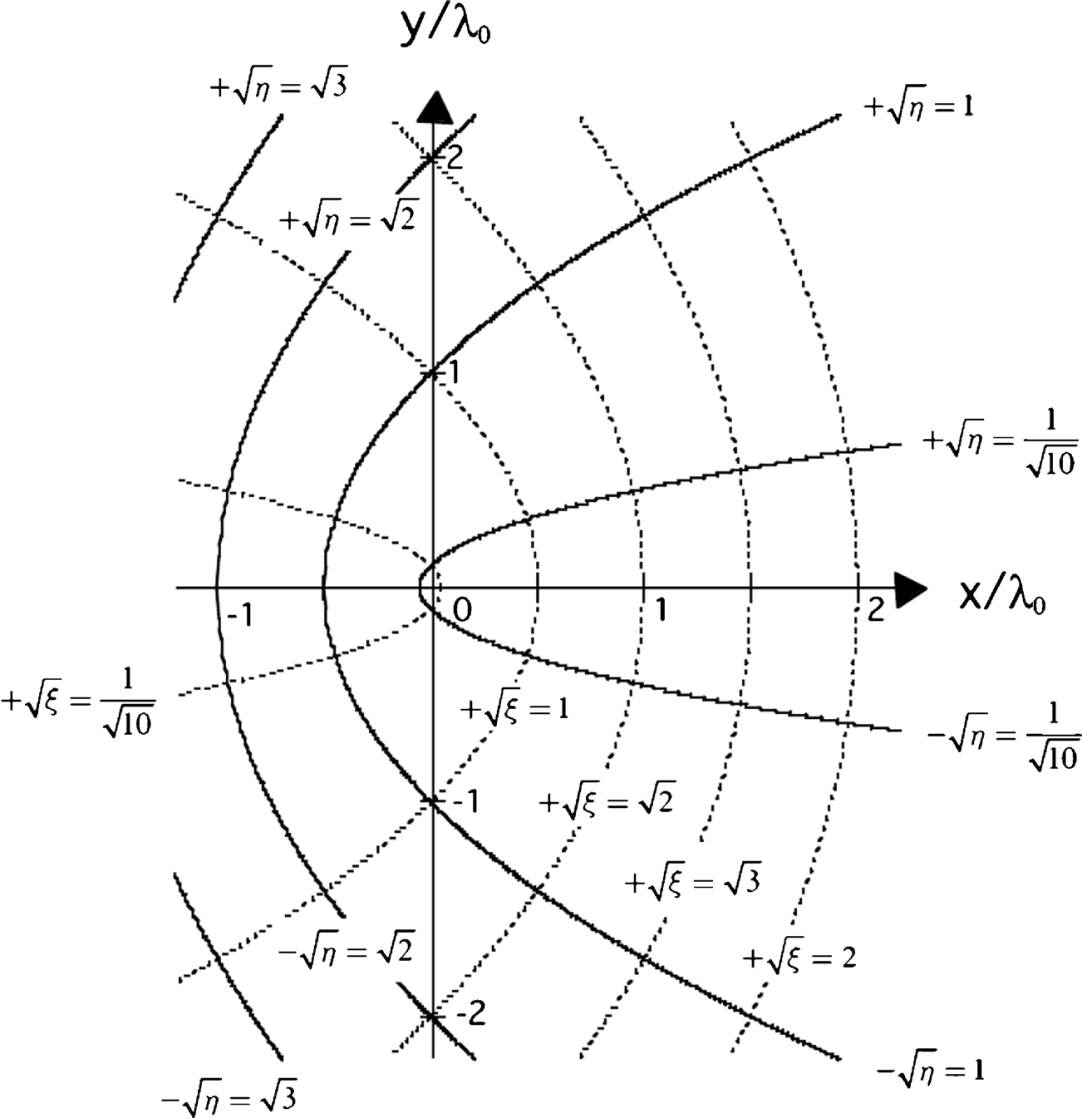
Projection of parabolic cylindrical coordinates onto the *x*-*y* plane

We consider two parabolic cylinder structures: a metallic parabolic cylinder surrounded by a dielectric and a dielectric parabolic cylinder surrounded by metal. In this paper, these are simply called a parabolic metal wedge and a parabolic metal groove, respectively. As shown in Fig. 1, the parabolic cylindrical coordinates (*ξ*, *η*, *z*) can be used to describe an infinite parabolic cylinder with permittivity *ε*_1_, and surrounded by matter with permittivity *ε*_2_. The parabolic cylindrical coordinates are related to the Cartesian coordinates (*x*, *y*, *z*) according to the following transformations [36–38]:

1 $$ x={2}^{-1}{\lambda}_0\left(\xi -\eta \right),y=\pm {\lambda}_0\sqrt{\xi \eta },z=z, $$

where *λ*_0_ is the wavelength of interest in vacuum used for a scale factor. We here choose for the *y*-coordinate of (1) to take $\sqrt{\xi }$ as positive; we must then take $\sqrt{\eta }$ with the positive sign on the semi-parabola above the *x*-axis and with the negative sign on the lower semi-parabola (see Fig. 1). Curves of constant $\sqrt{\xi }$ and $\sqrt{\eta }$ in the cross-section of the *x*-*y* plane for (1) are shown in Fig. 2, where all of the parabolas that open in the direction of the negative *x*-axis are full parabolas to which a single value of $\sqrt{\xi }$ within the interval $0\le \sqrt{\xi }<\infty$, i.e., 0 ≤  *ξ*  <  ∞, is assigned, while the parabolas that open in the direction of the positive *x*-axis are regarded as consisting of two semi-parabolas: the upper one is described by $+\sqrt{\eta }$ and the lower one by $-\sqrt{\eta }$ so that $\sqrt{\eta }$ has the interval $-\infty <\sqrt{\eta }<+\infty$ [38]. Therefore, all the points in Fig. 2 are uniquely assigned to both *ξ* and $\sqrt{\eta }$, where 0 ≤ *ξ* < ∞ and $-\infty <\sqrt{\eta }<+\infty$. For the parabola of Fig. 1, the upper and lower semi-parabolas are indicated by $+\sqrt{\eta }=\sqrt{\eta_0}$ and $-\sqrt{\eta }=\sqrt{\eta_0}$, respectively. The vertex point of the parabola in Fig. 1 is indicated by *x*/*λ*_0_ = − *η*_0_/2 on the *x*-axis. From the formula for the curvature of space curves (for examples, see Section 9.1.2–6 in [39]), we see that the radius of curvature, *ρ*_0_, at the apex point of the parabola has the relation

2 $$ {\rho}_0/{\lambda}_0={\eta}_0. $$

In the case where the imaginary parts of the permittivities are ignored, we have the conditions *ε*_1_ < 0, *ε*_2_ > 0 for the parabolic metal wedge and the conditions *ε*_1_ > 0, *ε*_2_ < 0 for the parabolic metal groove. Here, we examine simple plasmonic modes with translational symmetry about the *z*-axis, whose magnetic field at time *t* at the point located by the coordinate vector ***x*** is **H**(**x**, *t*) directed along the *z*-axis and depend on the intervals *ξ* ∈ [0, ∞) and $\sqrt{\eta}\in \left(-\infty, \infty \right)$; therefore, it is written as

3 $$ \mathbf{H}\left(\mathbf{x},t\right)=\left(0,0,{H}_z\left(\xi, \sqrt{\eta },t\right)\right) $$

in the parabolic cylindrical coordinate system. Assuming the harmonic time dependence *e* ^− *iωt*^, we can write

4 $$ {H}_z\left(\xi, \sqrt{\eta },t\right)=\mathrm{R}\mathrm{e}\left[{H}_z\left(\xi, \sqrt{\eta}\right){e}^{-i\omega t}\right], $$

where *ω* is the angular frequency of interest. Combining the two Maxwell curl equations without any sources, we find the wave equation for the magnetic field in (4):

5 $$ \frac{4\sqrt{\xi }}{\xi +\eta }{\partial}_{\xi}\left[\sqrt{\xi }{\partial}_{\xi }{H}_z\left(\xi, \sqrt{\eta}\right)\right]+\frac{4\sqrt{\eta }}{\xi +\eta }{\partial}_{\eta}\left[\sqrt{\eta }{\partial}_{\eta }{H}_z\left(\xi, \sqrt{\eta}\right)\right]+{\varepsilon}_j{\lambda}_0^2\frac{\omega^2}{c^2}{H}_z\left(\xi, \sqrt{\eta}\right)=0,j=1,2, $$

where *c* is the velocity of light in vacuum. After using the relation *λ*_0_ *ω*/*c* = 2*π*, Eq. (5) can be rewritten as [37]

6 $$ {\partial}_{\sqrt{\xi}\sqrt{\xi }}{H}_{zj}\left(\xi, \sqrt{\eta}\right)+{\partial}_{\sqrt{\eta}\sqrt{\eta }}{H}_{zj}\left(\xi, \sqrt{\eta}\right)+4{\pi}^2{\varepsilon}_j\left[{\left(\sqrt{\xi}\right)}^2+{\left(\sqrt{\eta}\right)}^2\right]{H}_{zj}\left(\xi, \sqrt{\eta}\right)=0,j=1,2 $$

with the definition of the magnetic field ${H}_z\left(\xi, \sqrt{\eta}\right)$ in the regions of permittivities *ε*_1_ and *ε*_2_ as ${H}_{z1}\left(\xi, \sqrt{\eta}\right)$ and ${H}_{z2}\left(\xi, \sqrt{\eta}\right)$, respectively. In (6), the magnetic field may be classified into even (+) or odd (−) symmetries with respect to $\sqrt{\eta }$, because the geometrical structure in Fig. 1 is invariant for the reflection in the *η* = 0 plane $\left(\sqrt{\xi}\to \sqrt{\xi },\sqrt{\eta}\to -\sqrt{\eta },z\to z\right)$ and the differential operator of the wave Eq. (6) is even under its reflection transformation. Accordingly, we express the magnetic field for even (+) and odd (−) symmetries such as ${H}_z^{+}\left(\xi, \sqrt{\eta}\right)$ and ${H}_z^{-}\left(\xi, \sqrt{\eta}\right)$, respectively. As in previous papers [13–15], here we look for solutions in the following form by using quasi-separation of variables:

7 $$ {H}_{zj}^s\left(\xi, \sqrt{\eta}\right)={\varXi}_j^s\left(\xi \right){H}_j^s\left(\sqrt{\eta },\xi \right),j=1,2fors=+,- $$

with the boundary conditions

8 $$ {H}_j^s\left(\sqrt{\eta_0},\xi \right)=1,j=1,2 $$

to determine *Η*_*j*_^*s*^(*η*, *ξ*) uniquely, where *s* indicates either the even (+) or odd (−) symmetry with respect to $\sqrt{\eta }$, i.e.,

9 $$ {H}_j^{\pm}\left(-\sqrt{\eta },\xi \right)=\pm {H}_j^{\pm}\left(\sqrt{\eta },\xi \right),j=1,2 $$

(double-sign corresponds). Substituting (7) into (6), we get for *j* = 1, 2 that

10 $$ \begin{array}{l}-\left[\frac{1}{\varXi_j^s\left(\xi \right)}{\partial}_{\sqrt{\xi}\sqrt{\xi }}{\varXi}_j^s\left(\xi \right)+4{\pi}^2{\varepsilon}_j{\left(\sqrt{\xi}\right)}^2\right]=\\ {}\left\{\begin{array}{l}\frac{1}{H_j^s\left(\sqrt{\eta },\xi \right)}{\partial}_{\sqrt{\eta}\sqrt{\eta }}{H}_j^s\left(\sqrt{\eta },\xi \right)+4{\pi}^2{\varepsilon}_j{\left(\sqrt{\eta}\right)}^2+\frac{1}{H_j^s\left(\sqrt{\eta },\xi \right)}{\partial}_{\sqrt{\xi}\sqrt{\xi }}{H}_j^s\left(\sqrt{\eta },\xi \right)\\ {}+\frac{2}{\varXi_j^s\left(\xi \right){H}_j^s\left(\sqrt{\eta },\xi \right)}\left[{\partial}_{\sqrt{\xi }}{\varXi}_j^s\left(\xi \right)\right]{\partial}_{\sqrt{\xi }}{H}_j^s\left(\sqrt{\eta },\xi \right)\end{array}\right\}.\end{array} $$

Since the left side of (10) depends on *ξ* alone, while the right side depends on both *ξ* and *η*, both sides must depend on *ξ* alone. Setting both sides equal to *ζ*_*j*_(*ξ*) for *j* = 1, 2 and rearranging terms in each equation, we obtain the radial equations

11 $$ {\partial}_{\sqrt{\xi}\sqrt{\xi }}{\varXi}_j^s\left(\xi \right)+\left[{\zeta}_j^s\left(\xi \right)+4{\pi}^2{\varepsilon}_j{\left(\sqrt{\xi}\right)}^2\right]{\varXi}_j^s\left(\xi \right)=0 $$

and the angular equations

12 $$ {\partial}_{\sqrt{\eta}\sqrt{\eta }}{H}_j^s\left(\sqrt{\eta },\xi \right)+\left[-{\zeta}_j^s\left(\xi \right)+4{\pi}^2{\varepsilon}_j{\left(\sqrt{\eta}\right)}^2\right]{H}_j^s\left(\sqrt{\eta },\xi \right)=-{F}_j^s\left(\sqrt{\eta },\xi \right) $$

where

13 $$ \begin{array}{l}{F}_j^s\left(\sqrt{\eta },\xi \right)={\partial}_{\sqrt{\xi}\sqrt{\xi }}{H}_j^s\left(\sqrt{\eta },\xi \right)+2{\left\{{\varXi}_j^s\left(\xi \right)\right\}}^{-1}\left[{\partial}_{\sqrt{\xi }}{\varXi}_j^s\left(\xi \right)\right]{\partial}_{\sqrt{\xi }}{H}_j^s\left(\sqrt{\eta },\xi \right)\\ {}\equiv {G}_j^s\left({H}_j^s\left(\sqrt{\eta },\xi \right),{\varXi}_j^s\left(\xi \right)\right).\end{array} $$

Here, *ζ*_*j*_^*s*^(*ξ*) for *j* = 1, 2 are called quasi-separation invariants, or simply separation quantities, which are analogous to the separation constants in classical separation of variables (CSOV). Now, we can replace (6) with (11) and (12), which satisfy the boundary conditions of (8).

## Unification of the Radial Equations in the Two Regions

The radial equations (11) are separately expressed in two different regions *j* = 1, 2. Remarkably, those equations can be transformed into a unified form independent of the regions by considering the limiting cases *ξ* → 0 + and *ξ* → ∞.

For *ξ* → 0 +, since the conditions, $\left|{\zeta}_j^s(0)\right|\gg 4{\pi}^2\left|{\varepsilon}_j\right|{\left(\sqrt{\xi}\right)}^2$, are acceptable unless *ζ*_*j*_(0) = 0, Eq. (11) become

14 $$ {\partial}_{\sqrt{\xi}\sqrt{\xi }}{\varXi}_j^s\left(\xi \right)+{\zeta}_j^s(0){\varXi}_j^s\left(\xi \right)=0\left(\xi \to 0+\right), $$

in which the material terms *ε*_*j*_ are lost and therefore the different expressions in the regions *j* = 1, 2 are a superfluity of equations. Then, by setting *ζ*_*u*_^*s*^(0) = *ζ*_*j*_^*s*^(0) and *Ξ*_*u*_^*s*^(*ξ*) = *Ξ*_*j*_^*s*^(*ξ*) for *ξ* → 0 +, we describe (14) as

15 $$ {\partial}_{\sqrt{\xi}\sqrt{\xi }}{\varXi}_u^s\left(\xi \right)+{\zeta}_u^s(0){\varXi}_u^s\left(\xi \right)=0\left(\xi \to 0+\right) $$

in the unified notation for the two different regions. Equation (15) is used to investigate asymptotic solutions for *Ξ*_*u*_^*s*^(*ξ*) as *ξ* → 0 +. Two nontrivial linearly independent particular solutions to (15) are given by

16 $$ {\varXi}_u^s\left(\xi \right)= \exp \left(i\nu \sqrt{\xi}\right), \exp \left(-i\nu \sqrt{\xi}\right) $$

with the definition $\nu =\sqrt{\zeta_u^s(0)}$. Remembering the time dependence *e* ^− *iωt*^ in (4), we see that $\exp \left(i\nu \sqrt{\xi}\right)$ and $\exp \left(-i\nu \sqrt{\xi}\right)$ in (16) correspond to the outgoing and incoming waves, respectively (the use of this condition may seem strange for a discussion of the condition *ξ* → 0 +), but it is very useful for characterization as seen in “Boundary Conditions for the Radial and Extended Angular Functions” section for *ξ* → ∞ when *ν* > 0.

For *ξ* → ∞, since the physical situation is considered the same as for SPPs in planar geometry, (11) can be described with a unified notation for the two regions *j* = 1, 2 in the form *Ξ*_*u*_^*s*^(*ξ*) = *Ξ*_*j*_^*s*^(*ξ*) and this unified radial function satisfies the Sommerfeld radiation conditions [40]:

17 $$ \underset{r\to \infty }{ \lim }r\left({\partial}_r{\varXi}_u^s\pm i{k}_p{\varXi}_u^s\right)=0 $$

where $r=\sqrt{x^2+{y}^2}$, and *k*_*p*_ is the wave number of SPPs in the planar geometry, given [41, 42] by ${k}_p={k}_0\sqrt{\varepsilon_1{\varepsilon}_2/\left({\varepsilon}_1+{\varepsilon}_2\right)}$ with the wave number in vacuum, *k*_0_, defined by *k*_0_ = *ω*/*c*. Using (1), we can transform (17) into the form

18 $$ \underset{\xi \to \infty }{ \lim}\left(\xi +\eta \right)\left(2{\lambda_0}^{-1}{\partial}_{\xi}\pm i{k}_p\right){\varXi}_u^s\left(\xi \right)=0. $$

This equation allows us to choose simple candidates for the unified radial function in the form, *Ξ*_*u*_^*s*^(*ξ*) = exp(±*iλ*_0_ *k*_*p*_ *ξ*/2) for *ξ* → ∞, which, unfortunately, do not belong to a class of solutions for (11). Then, carefully choosing for (18) that *Ξ*_*u*_^*s*^(*ξ*) = *ξ* ^− 1/4^ exp(±*iλ*_0_ *k*_*p*_ *ξ*/2) for *ξ* → ∞, we obtain their differential equation

19 $$ {\partial}_{\sqrt{\xi}\sqrt{\xi }}{\varXi}_u^s\left(\xi \right)+\left({\lambda}_0^2{k}_p^2{\left(\sqrt{\xi}\right)}^2-3/4{\left(\sqrt{\xi}\right)}^2\right){\varXi}_u^s\left(\xi \right)=0\left(\xi \to \infty \right) $$

or

20 $$ {\partial}_{\sqrt{\xi}\sqrt{\xi }}{\varXi}_u^s\left(\xi \right)+{\lambda}_0^2{k}_p^2{\left(\sqrt{\xi}\right)}^2{\varXi}_u^s\left(\xi \right)=0\left(\xi \to \infty \right) $$

under the acceptable condition, ${\lambda}_0^2{k}_p^2{\left(\sqrt{\xi}\right)}^2\gg 3/4{\left(\sqrt{\xi}\right)}^2$ for *ξ* → ∞. Since (20) can be derived from (11) by using the unified notation of *Ξ*_*u*_^*s*^(*ξ*) = *Ξ*_*j*_^*s*^(*ξ*) and setting ${\zeta}_j^s\left(\xi \right)={\lambda}_0^2{\beta}_j^2{\left(\sqrt{\xi}\right)}^2$ with ${\beta}_j=\sqrt{k_p^2-{\varepsilon}_j{k}_0^2}$ for *j* = 1, 2, Eq. (20) is regarded as the limiting equation of (11) for *ξ* → ∞.

Now, we are ready to look for a unified form of (11) by following an inversion process of finding the limiting Eqs. (15) and (20) as *ξ* → 0 + and *ξ* → ∞, respectively. A simple candidate for the unified radial equation is given by

21 $$ {\partial}_{\sqrt{\xi}\sqrt{\xi }}{\varXi}_u^s\left(\xi \right)+\left[{\lambda}_0^2{k}_p^2{\left(\sqrt{\xi}\right)}^2+{\zeta}_u^s(0)\right]{\varXi}_u^s\left(\xi \right)=0\left(0\le \xi <\infty \right), $$

which approaches (15) and (20) as *ξ* → 0 + and *ξ* → ∞, respectively. Then, we can find without difficulty that more general candidate is given by

22 $$ {\partial}_{\sqrt{\xi}\sqrt{\xi }}{\varXi}_u^s\left(\xi \right)+\left[{\lambda}_0^2{k}_p^2{\left(\sqrt{\xi}\right)}^2+{\zeta}_u^s(0)+{A}^s\left(\xi \right)\right]{\varXi}_u^s\left(\xi \right)=0\left(0\le \xi <\infty \right) $$

where *A* ^*s*^(*ξ*) is arbitrary only if *A* ^*s*^(*ξ*) → 0 as *ξ* → 0 and *A* ^*s*^(*ξ*)/*ξ* → 0 as *ξ* → ∞. By setting *A* ^*s*^(*ξ*) = *ζ*_*u*_^*s*^(*ξ*) − *ζ*_*u*_^*s*^(0), we can rewrite (22) as

23 $$ {\partial}_{\sqrt{\xi}\sqrt{\xi }}{\varXi}_u^s\left(\xi \right)+\left[{\lambda}_0^2{k}_p^2{\left(\sqrt{\xi}\right)}^2+{\zeta}_u^s\left(\xi \right)\right]{\varXi}_u^s\left(\xi \right)=0\left(0\le \xi <\infty \right), $$

where *ζ*_*u*_^*s*^(*ξ*) satisfies the condition *ζ*_*u*_^*s*^(*ξ*)/*ξ* → 0 as *ξ* → ∞. Comparing (23) with (11), we find that the unification conditions are given by

24 $$ {\zeta}_j^s\left(\xi \right)={\zeta}_u^s\left(\xi \right)+{\lambda}_0^2{\beta}_j^2{\left(\sqrt{\xi}\right)}^2,j=1,2\left(0\le \xi <\infty \right), $$

which allows for the unified notation of the radial functions as follows:

25 $$ {\varXi}_u^s\left(\xi \right)={\varXi}_j^s\left(\xi \right),j=1,2\left(0\le \xi <\infty \right). $$

Further discussion on the unified radial Eq. (23) would require more detailed information on the unified separation quantity *ζ*_*u*_^*s*^(*ξ*), which can be determined from the boundary conditions.

## Boundary Conditions for the Radial and Extended Angular Functions

In the preceding section, we showed that original radial Eq. (11) can be simplified into the unified radial Eq. (23), in which the unified radial function for *ξ* → 0 + can be expressed by a linear combination of the outgoing wave, $\exp \left(i\nu \sqrt{\xi}\right)$, and the incoming wave, $\exp \left(-i\nu \sqrt{\xi}\right)$, as seen in (16). In this case, the unified radial function *Ξ*_*u*_^*s*^(*ξ*) is divided into an incoming part, *Ξ*_*in*_^*s*^(*ξ*), and an outgoing part, *Ξ*_*out*_^*s*^(*ξ*); that is

26 $$ {\varXi}_u^s\left(\xi \right)={\varXi}_{in}^s\left(\xi \right)+{\varXi}_{out}^s\left(\xi \right), $$

which satisfies the boundary condition

27 $$ \underset{\xi \to 0+}{ \lim }{\varXi}_{in}^s\left(\xi \right)=\underset{\xi \to 0+}{ \lim }{\varXi}_{out}^s\left(\xi \right). $$

By choosing

28 $$ {\varXi}_{in}^s\left(\xi \right)\sim {H}_0 \exp \left(-i\nu \sqrt{\xi}\right),{\varXi}_{out}^s\left(\xi \right)\sim {H}_0 \exp \left(i\nu \sqrt{\xi}\right)\left(\xi \to 0+\right) $$

and substituting (28) into (26), we obtain the boundary condition of the unified radial function as follows:

29 $$ {\varXi}_u^s\left(\xi \right)\sim 2{H}_0 \cosh \left(i\nu \sqrt{\xi}\right)=2{H}_0+O\left(\xi \right)\left(\xi \to 0+\right), $$

where *H*_0_ is the complex amplitude of the magnetic field to be determined by the initial conditions.

In order to obtain the boundary condition of the angular functions, we can use the continuity of the radial electric field at the metal-dielectric surface ($+\sqrt{\eta }=\sqrt{\eta_0}$). The Ampére-Maxwell equation in the absence of current density allows us to describe the electric field as a function of the magnetic filed. Since the magnetic field is given by (3), the electric field ***E***(***x***, *t*) can be described as

30 $$ E\left(x,t\right)=\left({E}_{\xi}\left(\xi, \sqrt{\eta },t\right),{E}_{\eta}\left(\xi, \sqrt{\eta },t\right),0\right) $$

in which we see from (4) that

31 $$ {E}_{\xi}\left(\xi, \sqrt{\eta },t\right)=\mathrm{R}\mathrm{e}\left[{E}_{\xi}\left(\xi, \sqrt{\eta}\right){e}^{-i\omega t}\right],{E}_{\eta}\left(\xi, \sqrt{\eta },t\right)=\mathrm{R}\mathrm{e}\left[{E}_{\eta}\left(\xi, \sqrt{\eta}\right){e}^{-i\omega t}\right]. $$

Each component of the electric field is described by using the magnetic field as

32 $$ {E}_{\xi j}^s\left(\xi, \sqrt{\eta}\right)=i{\left(\pi {\varepsilon}_j\right)}^{-1}\sqrt{\eta /\left(\xi +\eta \right)}{\partial}_{\eta }{H}_{zj}^s\left(\xi, \sqrt{\eta}\right) $$

33 $$ {E}_{\eta j}^s\left(\xi, \sqrt{\eta}\right)=-i{\left(\pi {\varepsilon}_j\right)}^{-1}\sqrt{\xi /\left(\xi +\eta \right)}{\partial}_{\xi }{H}_{zj}^s\left(\xi, \sqrt{\eta}\right), $$

where the superscript *j* = 1, 2 indicates the region of permittivities *ε*_1_ and *ε*_2_, respectively. Substituting (7) into (32), and using (25), we can rewrite the radial electric field for *j* = 1, 2 as

34 $$ {E}_{\xi j}^s\left(\xi, \sqrt{\eta}\right)=i{\left(2\pi {\varepsilon}_j\sqrt{\xi +\eta}\right)}^{-1}{\varXi}_u^s\left(\xi \right){\partial}_{\sqrt{\eta }}{H}_j^s\left(\sqrt{\eta },\xi \right) $$

which is continuous at the metal-dielectric surface ($+\sqrt{\eta }=\sqrt{\eta_0}$). Hence, we find the following expression for the boundary conditions of the angular functions:

35 $$ {\varepsilon_1}^{-1}\underset{\eta \to {\eta}_0-0}{ \lim }{\partial}_{\sqrt{\eta }}{H}_1^s\left(\sqrt{\eta },\xi \right)={\varepsilon_2}^{-1}\underset{\eta \to {\eta}_0+0}{ \lim }{\partial}_{\sqrt{\eta }}{H}_2^s\left(\sqrt{\eta },\xi \right). $$

Note that the continuity of the radial electric field on the other metal-dielectric surface ($-\sqrt{\eta }=\sqrt{\eta_0}$) is automatically satisfied when the magnetic field has either even (+) or odd (−) symmetries with respect to $\sqrt{\eta }$.

## Application of Perturbation Methods for Solving the Extended Angular Equations

According to (24) and (25), the extended angular Eqs. (12) and (13) for *j* = 1, 2 can be simplified to the forms

36 $$ {\partial}_{\sqrt{\eta}\sqrt{\eta }}{H}_j^s\left(\sqrt{\eta },\xi \right)+\left[-{\zeta}_u^s\left(\xi \right)-{\lambda}_0^2{\beta}_j^2{\left(\sqrt{\xi}\right)}^2+4{\pi}^2{\varepsilon}_j{\left(\sqrt{\eta}\right)}^2\right]\times {H}_j^s\left(\sqrt{\eta },\xi \right)=-{F}_j^s\left(\sqrt{\eta },\xi \right) $$

37 $$ {F}_j^s\left(\sqrt{\eta },\xi \right)={G}_j^s\left({H}_j^s\left(\sqrt{\eta },\xi \right),{\varXi}_u^s\left(\xi \right)\right). $$

In spite of this simplification, we still face difficulty with the fact that (36) and (37) include the as-yet-to-be-determined unified radial function *Ξ*_*u*_^*s*^(*ξ*). Moreover, we still face a basic difficulty in solving such a complicated partial differential equation (PDE) containing first- and second-order partial derivatives with respect to $\sqrt{\xi }$ and a second-order partial derivatives with respect to $\sqrt{\eta }$. To overcome these difficulties, we apply perturbation methods to the extended angular Eq. (36) by treating ${F}_j^s\left(\sqrt{\eta },\xi \right)$ on the right side as a perturbing term because an exact solution can be found for the left side. According to the perturbation theory [43], we introduce the perturbation parameter 0 ≤ *δ* ≤ 1 into (36) by replacing ${F}_j^s\left(\sqrt{\eta },\xi \right)$ with $\delta {F}_j^s\left(\sqrt{\eta },\xi \right)$ on the right side and look for solutions of the perturbed equation of the form

38 $$ {H}_j^s\left(\sqrt{\eta },\xi \right)={H}_j^{s(0)}\left(\sqrt{\eta },\xi \right)+\delta {H}_j^{s(1)}\left(\sqrt{\eta },\xi \right)+\cdot \cdot \cdot, j=1,2. $$

Accordingly, *ζ*_*u*_^*s*^(*ξ*) and *Ξ*_*u*_^*s*^(*ξ*) should be described as

39 $$ {\zeta}_u^s\left(\xi \right)={\zeta}_u^{s(0)}\left(\xi \right)+\delta {\zeta}_u^{s(1)}\left(\xi \right)+\cdot \cdot \cdot $$

40 $$ {\varXi}_u^s\left(\xi \right)={\varXi}_u^{s(0)}\left(\xi \right)+\delta {\varXi}_u^{s(1)}\left(\xi \right)+\cdot \cdot \cdot, $$

respectively. For *Ξ*_*u*_(*ξ*), the incoming *Ξ*_*in*_(*ξ*) and the outgoing *Ξ*_*out*_(*ξ*) should also be described as

41 $$ {\varXi}_{in}^s\left(\xi \right)={\varXi}_{in}^{s(0)}\left(\xi \right)+\delta {\varXi}_{in}^{s(1)}\left(\xi \right)+\cdot \cdot \cdot $$

42 $$ {\varXi}_{out}^s\left(\xi \right)={\varXi}_{out}^{s(0)}\left(\xi \right)+\delta {\varXi}_{out}^{s(1)}\left(\xi \right)+\cdot \cdot \cdot, $$

respectively. Substituting (38)–(40) into the perturbed equation, and setting the coefficients of the powers of *δ* equal to each other, we have a system of equations for ${H}_j^{s(0)}\left(\sqrt{\eta },\xi \right)$, ${H}_j^{s(1)}\left(\sqrt{\eta },\xi \right)$,…, in the power series of (38); for coefficient of *δ* ^0^, we have

43 $$ {\partial}_{\sqrt{\eta}\sqrt{\eta }}{H}_j^{s(0)}\left(\sqrt{\eta },\xi \right)+\left[-{\zeta}_u^{s(0)}\left(\xi \right)-{\lambda}_0^2{\beta}_j^2{\left(\sqrt{\xi}\right)}^2+4{\pi}^2{\varepsilon}_j{\left(\sqrt{\eta}\right)}^2\right]\times {H}_j^{s(0)}\left(\sqrt{\eta },\xi \right)=0. $$

Setting $\sqrt{\eta }=\sqrt{\eta_0}$ in (38) and using (8), we obtain for *j* = 1, 2 that

44 $$ {H}_j^s\left(\sqrt{\eta_0},\xi \right)={H}_j^{s(0)}\left(\sqrt{\eta_0},\xi \right)+\delta {H}_j^{s(1)}\left(\sqrt{\eta_0},\xi \right)+\cdot \cdot \cdot =1, $$

which leads to the following Dirichlet boundary conditions

45 $$ {H}_j^{s(0)}\left(\sqrt{\eta_0},\xi \right)=1,{H}_j^{s(1)}\left(\sqrt{\eta_0},\xi \right)={H}_j^{s(2)}\left(\sqrt{\eta_0},\xi \right)=\cdot \cdot \cdot =0 $$

by setting the coefficients of the powers of *δ* equal to each other in (44). In a similar manner, by substituting (38) into (35), we obtain the following Neumann boundary conditions

46 $$ {\varepsilon_1}^{-1}\underset{\eta \to {\eta}_0-0}{ \lim }{\partial}_{\sqrt{\eta }}{H}_1^{s(n)}\left(\sqrt{\eta },\xi \right)={\varepsilon_2}^{-1}\underset{\eta \to {\eta}_0+0}{ \lim }{\partial}_{\sqrt{\eta }}{H}_2^{s(n)}\left(\sqrt{\eta },\xi \right),n=0,1,2,.... $$

Note that the imposition of both the Dirichlet (45) and Neumann boundary conditions (46) is referred to as the Cauchy boundary conditions [44]; that is, we specify the values and normal derivatives of the functions ${H}_j^{s(0)}\left(\sqrt{\eta },\xi \right)$, ${H}_j^{s(1)}\left(\sqrt{\eta },\xi \right)$,…, in the power series (38) along the metal-dielectric boundary ($\sqrt{\eta }=\sqrt{\eta_0}$).

Perturbation methods should also be applied to the unified radial Eq. (23). Substituting (39) and (40) into the unified radial Eq. (23) and equating the coefficients of like powers of *δ* on both sides, we have a system of equations for *Ξ*_*u*_^*s*(0)^(*ξ*), *Ξ*_*u*_^*s*(1)^(*ξ*),…, in the power series (40); for coefficient of *δ* ^0^, we have

47 $$ {\partial}_{\sqrt{\xi}\sqrt{\xi }}{\varXi}_u^{s(0)}\left(\xi \right)+\left[{\lambda}_0^2{k}_p^2{\left(\sqrt{\xi}\right)}^2+{\zeta}_u^{s(0)}\left(\xi \right)\right]{\varXi}_u^{s(0)}\left(\xi \right)=0\left(0\le \xi <\infty \right). $$

Substituting (40) into (29), and setting the coefficients of the powers of *δ* equal to each other, we have the following Dirichlet boundary conditions:

48 $$ {\varXi}_u^{s(0)}\left(\xi \right)\sim 2{H}_0,{\varXi}_u^{s(1)}\left(\xi \right)={\varXi}_u^{s(2)}\left(\xi \right)=\cdot \cdot \cdot =0\left(\xi \to 0+\right). $$

In a similar manner, by substituting (40)–(42) into (26), we have the following relations:

49 $$ {\varXi}_u^{s(n)}\left(\xi \right)={\varXi}_{in}^{s(n)}\left(\xi \right)+{\varXi}_{out}^{s(n)}\left(\xi \right),n=0,1,2,.... $$

Similarly, by substituting (41) and (42) into (27), we have the following Dirichlet boundary conditions:

50 $$ \underset{\xi \to 0+}{ \lim }{\varXi}_{in}^{s(n)}\left(\xi \right)=\underset{\xi \to 0+}{ \lim }{\varXi}_{out}^{s(n)}\left(\xi \right),n=0,1,2,.... $$

Summing up the points we have discussed in this section, we arrive at a sequence of problems, *P*_0_, *P*_1_, *P*_2_,…, from which we can find the function sets $\left\{{\zeta}_u^{s(n)}\left(\xi \right),{\varXi}_u^{s(n)}\left(\xi \right),{H}_1^{s(n)}\left(\eta, \xi \right),{H}_2^{s(n)}\left(\eta, \xi \right)\right\}$ for *n* = 0, 1, 2, … in sequence; the problem *P*_0_ is given by

51 $$ {P}_0\left\{\begin{array}{l}{\partial}_{\sqrt{\eta}\sqrt{\eta }}{H}_j^{s(0)}\left(\sqrt{\eta },\xi \right)+\left[-{\zeta}_u^{s(0)}\left(\xi \right)-{\lambda}_0^2{\beta}_j^2{\left(\sqrt{\xi}\right)}^2+4{\pi}^2{\varepsilon}_j{\left(\sqrt{\eta}\right)}^2\right]{H}_j^{s(0)}\left(\sqrt{\eta },\xi \right)=0,\hfill \\ {}\kern9em {H}_j^{s(0)}\left(\sqrt{\eta_0},\xi \right)=1forj=1,2\hfill \\ {}\kern4em {\varepsilon_1}^{-1}\underset{\eta \to {\eta}_0-0}{ \lim }{\partial}_{\sqrt{\eta }}{H}_1^{s(0)}\left(\sqrt{\eta },\xi \right)={\varepsilon_2}^{-1}\underset{\eta \to {\eta}_0+0}{ \lim }{\partial}_{\sqrt{\eta }}{H}_2^{s(0)}\left(\sqrt{\eta },\xi \right)\hfill \\ {}\kern4em {\partial}_{\sqrt{\xi}\sqrt{\xi }}{\varXi}_u^{s(0)}\left(\xi \right)+\left[{\lambda}_0^2{k}_p^2{\left(\sqrt{\xi}\right)}^2+{\zeta}_u^{s(0)}\left(\xi \right)\right]{\varXi}_u^{s(0)}\left(\xi \right)=0\hfill \\ {}{\left.\kern10em {\varXi}_u^{s(0)}\left(\xi \right)\right|}_{\xi \to 0+}=2{H}_0\hfill \\ {}\kern3em \underset{\xi \to 0+}{ \lim }{\varXi}_{in}^{s(0)}\left(\xi \right)=\underset{\xi \to 0+}{ \lim }{\varXi}_{out}^{s(0)}\left(\xi \right),{\varXi}_u^{s(0)}\left(\xi \right)={\varXi}_{in}^{s(0)}\left(\xi \right)+{\varXi}_{out}^{s(0)}\left(\xi \right).\hfill \end{array}\right. $$

The higher-order problems, *P*_1_, *P*_2_,…, are not shown here because they are not treated in this paper due to some difficulties involved in numerical calculations.

## Zeroth-Order Approximate Solutions for the Parabolic Metal Groove

### Extended Angular Functions of the Zeroth-Order for the Parabolic Metal Groove

The zeroth-order extended angular Eq. (43) are considered for the parabolic metal groove, in which we can write *ε*_1_ = *ε*_*d*_ and *ε*_2_ = *ε*_*m*_ by introducing notations of metallic permittivity *ε*_*m*_ < 0 and dielectric permittivity *ε*_*d*_ > 0. The solutions to (43) must be divided into even (+) and odd (−) symmetries with respect to $\sqrt{\eta }$, described as ${H}_j^{+(0)}\left(\sqrt{\eta },\xi \right)$ and ${H}_j^{-(0)}\left(\sqrt{\eta },\xi \right)$, respectively, for *j* = 1, 2.

Substituting *ε*_1_ = *ε*_*d*_(> 0) into the zeroth-order extended angular Eq. (43) for *j* = 1, we have

52 $$ {\partial}_{\sqrt{\eta}\sqrt{\eta }}{H}_1^{s(0)}\left(\sqrt{\eta },\xi \right)+\left[-{\zeta}_u^{s(0)}\left(\xi \right)-{\lambda}_0^2{\beta}_d^2{\left(\sqrt{\xi}\right)}^2+4{\pi}^2{\varepsilon}_d{\left(\sqrt{\eta}\right)}^2\right]\times {H}_1^{s(0)}\left(\sqrt{\eta },\xi \right)=0, $$

where ${\beta}_d=\sqrt{k_p^2-{\varepsilon}_d{k}_0^2}$. Dividing (52) by 4*πε*_*d*_^1/2^ and rearranging terms, we obtain

53 $$ \frac{\partial^2}{\partial {\left(2\sqrt{\pi {\varepsilon}_d^{1/2}\eta}\right)}^2}{H}_1^{s(0)}\left(\sqrt{\eta },\xi \right)+\left[\frac{1}{4}{\left(2\sqrt{\pi {\varepsilon}_d^{1/2}\eta}\right)}^2-\frac{\zeta_u^{s(0)}\left(\xi \right)+{\lambda}_0^2{\beta}_d^2\xi }{4\pi {\varepsilon}_d^{1/2}}\right]\times {H}_1^{s(0)}\left(\sqrt{\eta },\xi \right)=0, $$

which is a special case of the modified parabolic cylinder Eq. (A.7) corresponding to the parameter values

54 $$ z=2\sqrt{\pi {\varepsilon}_d^{1/2}\eta}\equiv {u}_d\left(\sqrt{\eta}\right),a=\left\{{\zeta}_u^{s(0)}\left(\xi \right)+{\lambda}_0^2{\beta}_d^2\xi \right\}/4\pi {\varepsilon}_d^{1/2}\equiv {v}_d^s\left(\xi \right). $$

Since the solutions to (53) must be divided into even (+) and odd (−) symmetries with respect to $\sqrt{\eta }$, written as ${H}_1^{+(0)}\left(\sqrt{\eta },\xi \right)$ and ${H}_1^{-(0)}\left(\sqrt{\eta },\xi \right)$, respectively, by taking into account the boundary conditions ${H}_1^{s(0)}\left(\sqrt{\eta_0},\xi \right)=1$ for *s* =+, − in (51), we can describe the solutions to (53) as

55 $$ {H}_1^{+(0)}\left(\sqrt{\eta },\xi \right)={w}_1\left({v}_d^{+}\left(\xi \right),{u}_d\left(\sqrt{\eta}\right)\right)/{w}_1\left({v}_d^{+}\left(\xi \right),{u}_d\left(\sqrt{\eta_0}\right)\right) $$

56 $$ {H}_1^{-(0)}\left(\sqrt{\eta },\xi \right)={w}_2\left({v}_d^{-}\left(\xi \right),{u}_d\left(\sqrt{\eta}\right)\right)/{w}_2\left({v}_d^{-}\left(\xi \right),{u}_d\left(\sqrt{\eta_0}\right)\right), $$

where *w*_1_ and *w*_2_ are even and odd functions, respectively (see Appendix A).

In a similar way, by substituting *ε*_2_ = *ε*_*m*_(< 0) into (43) for *j* = 2, we have

57 $$ {\partial}_{\sqrt{\eta}\sqrt{\eta }}{H}_2^{s(0)}\left(\sqrt{\eta },\xi \right)+\left[-{\zeta}_u^{s(0)}\left(\xi \right)-{\lambda}_0^2{\beta}_m^2{\left(\sqrt{\xi}\right)}^2-4{\pi}^2\left|{\varepsilon}_m\right|{\left(\sqrt{\eta}\right)}^2\right]\times {H}_2^{s(0)}\left(\sqrt{\eta },\xi \right)=0, $$

where ${\beta}_m=\sqrt{k_p^2-{\varepsilon}_m{k}_0^2}$. Dividing (57) by 4*π*|*ε*_*m*_|^1/2^ and rearranging terms, we obtain

58 $$ \frac{\partial^2}{\partial {\left(2\sqrt{\pi {\left|{\varepsilon}_m\right|}^{1/2}\eta}\right)}^2}{H}_2^{s(0)}\left(\sqrt{\eta },\xi \right)-\left[\frac{1}{4}{\left(2\sqrt{\pi {\left|{\varepsilon}_m\right|}^{1/2}\eta}\right)}^2+\frac{\zeta_u^{s(0)}\left(\xi \right)+{\lambda}_0^2{\beta}_m^2\xi }{4\pi {\left|{\varepsilon}_m\right|}^{1/2}}\right]{H}_2^{s(0)}\left(\sqrt{\eta },\xi \right)=0, $$

which is a special case of the parabolic cylinder Eq. (A.1) corresponding to the parameter values

59 $$ z=2\sqrt{\pi {\left|{\varepsilon}_m\right|}^{1/2}\eta}\equiv {u}_m\left(\sqrt{\eta}\right),a=\left\{{\zeta}_u^{s(0)}\left(\xi \right)+{\lambda}_0^2{\beta}_m^2\xi \right\}/4\pi {\left|{\varepsilon}_m\right|}^{1/2}\equiv {v}_m^s\left(\xi \right). $$

Since ${H}_2^{s(0)}\left(\sqrt{\eta },\xi \right)$ for *s* =+, − tends to zero as $\sqrt{\eta}\to \pm \infty$ and must satisfy the boundary conditions ${H}_2^{s(0)}\left(\sqrt{\eta_0},\xi \right)=1$ for *s* =+, − in (51), by taking into account (A.2) and (A.3) for asymptotic behavior at infinity, we can describe solutions of (58) for $\sqrt{\eta}\in \left[\sqrt{\eta_0},\infty \right)$ as

60 $$ {H}_2^{s(0)}\left(\sqrt{\eta },\xi \right)=U\left({v}_m^s\left(\xi \right),{u}_m\left(\sqrt{\eta}\right)\right)/U\left({v}_m^s\left(\xi \right),{u}_m\left(\sqrt{\eta_0}\right)\right) $$

for *s* =+, −. For $\sqrt{\eta}\in \left(-\infty, -\sqrt{\eta_0}\right]$, solutions to (58) can be easily obtained by using (60) through the relations of (9); they are not shown here explicitly.

### Characteristic Equations for Determining the Unified Separation Quantity of the Zeroth-Order for the Parabolic Metal Groove

By substituting ${H}_1^{+(0)}\left(\sqrt{\eta },\xi \right)$ in (55) and ${H}_2^{+(0)}\left(\sqrt{\eta },\xi \right)$ in (60) into the boundary condition for *s* = +, *ε*_1_ = *ε*_*d*_, and *ε*_2_ = *ε*_*m*_ in (35), we obtain

61 $$ \frac{1}{\varepsilon_d^{1/2}}\frac{{w_1}^{\prime}\left({v}_d^{+}\left(\xi \right),{u}_d\left(\sqrt{\eta_0}\right)\right)}{w_1\left({v}_d^{+}\left(\xi \right),{u}_d\left(\sqrt{\eta_0}\right)\right)}=-\frac{1}{{\left|{\varepsilon}_m\right|}^{1/2}}\frac{U^{\prime}\left({v}_m^{+}\left(\xi \right),{u}_m\left(\sqrt{\eta_0}\right)\right)}{U\left({v}_m^{+}\left(\xi \right),{u}_m\left(\sqrt{\eta_0}\right)\right)}, $$

where we use notations of ${w}_1\hbox{'}\left(a,z\right)={\partial}_z{w}_1\left(a,z\right)$ and *U* ^'^(*a*, *z*) = ∂_*z*_ *U*(*a*, *z*). Equation (61) is the characteristic equation for determining the zeroth-order unified separation quantity for the even (+) symmetry, *ζ*_*u*_^+ (0)^(*ξ*), in the parabolic metal groove. Similarly, by substituting ${H}_1^{-(0)}\left(\sqrt{\eta },\xi \right)$ in (56) and ${H}_2^{-(0)}\left(\sqrt{\eta },\xi \right)$ in (60) into the boundary condition for *s* = −, *ε*_1_ = *ε*_*d*_, and *ε*_2_ = *ε*_*m*_ in (35), we obtain

62 $$ \frac{1}{\varepsilon_d^{1/2}}\frac{{w_2}^{\prime}\left({v}_d^{-}\left(\xi \right),{u}_d\left(\sqrt{\eta_0}\right)\right)}{w_2\left({v}_d^{-}\left(\xi \right),{u}_d\left(\sqrt{\eta_0}\right)\right)}=-\frac{1}{{\left|{\varepsilon}_m\right|}^{1/2}}\frac{U^{\prime}\left({v}_m^{-}\left(\xi \right),{u}_m\left(\sqrt{\eta_0}\right)\right)}{U\left({v}_m^{-}\left(\xi \right),{u}_m\left(\sqrt{\eta_0}\right)\right)}, $$

where we use notation of *w*_2_^'^(*a*, *z*) = ∂*w*_2_(*a*, *z*)/∂*z*. Equation (62) is the characteristic equation for determining the zeroth-order unified separation quantity for the odd (−) symmetry, *ζ*_*u*_^− (0)^(*ξ*), in the parabolic metal groove.

### Approximate Determination of the Zeroth-Order Unified Separation Quantity Based on a Figure-Merit Function for the Parabolic Metal Groove

**Fig. 3:**
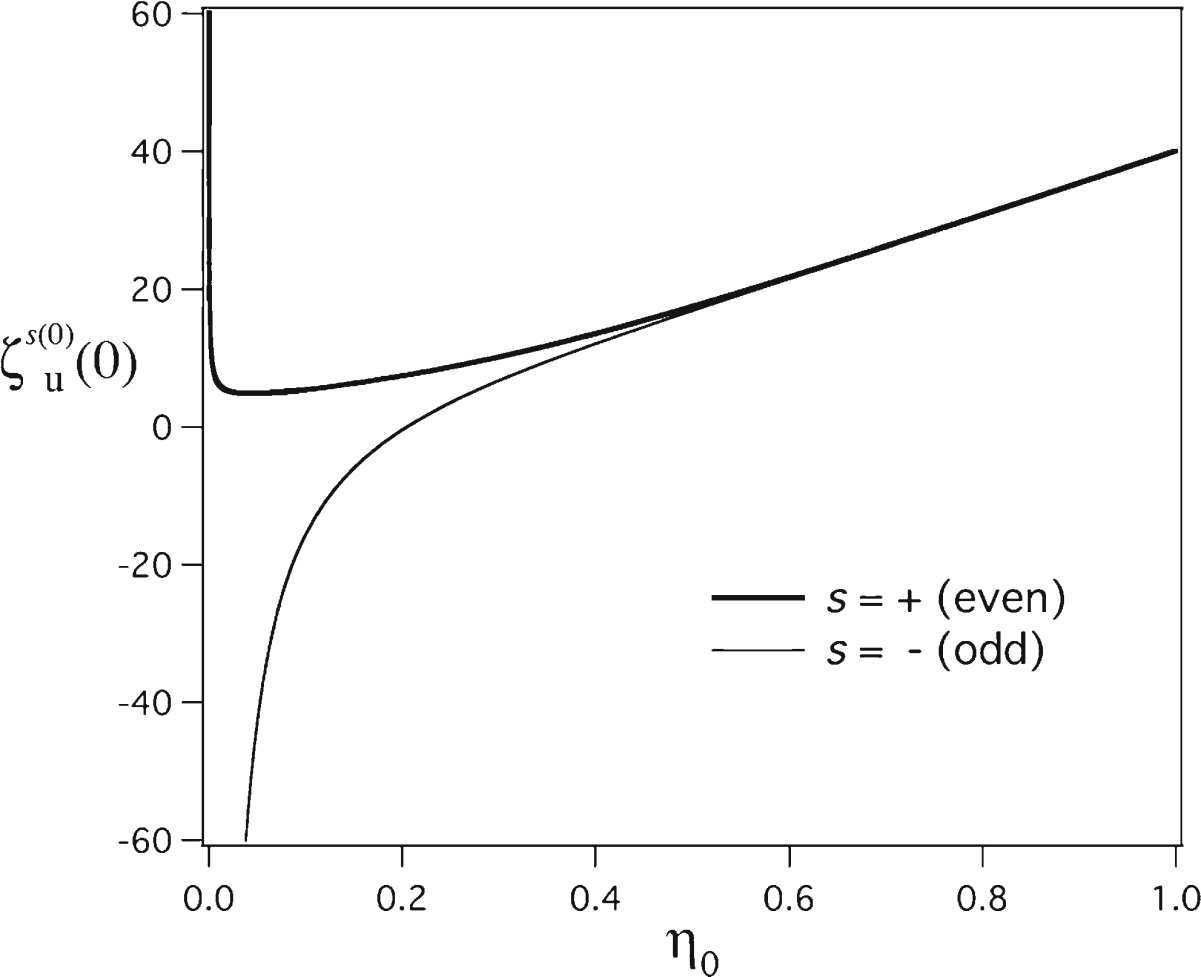
Numerical calculations of the even (+) and odd (−) zeroth-order unified separation quantities at *ξ* = 0, *ζ*_*u*_^+ (0)^(0), and *ζ*_*u*_^− (0)^(0), as a function of the parameter *η*_0_ for the parabolic surface $\pm \sqrt{\eta }=\sqrt{\eta_0}$ when *ξ* = 0, *ε*_*d*_ = 1, and *ε*_*m*_ = − 20 are used in (61) and (62), respectively. The specific value of *ζ*_*u*_^− (0)^(0) = 0 is obtained as *η*_0_ = 0.204785 numerically

**Fig. 4:**
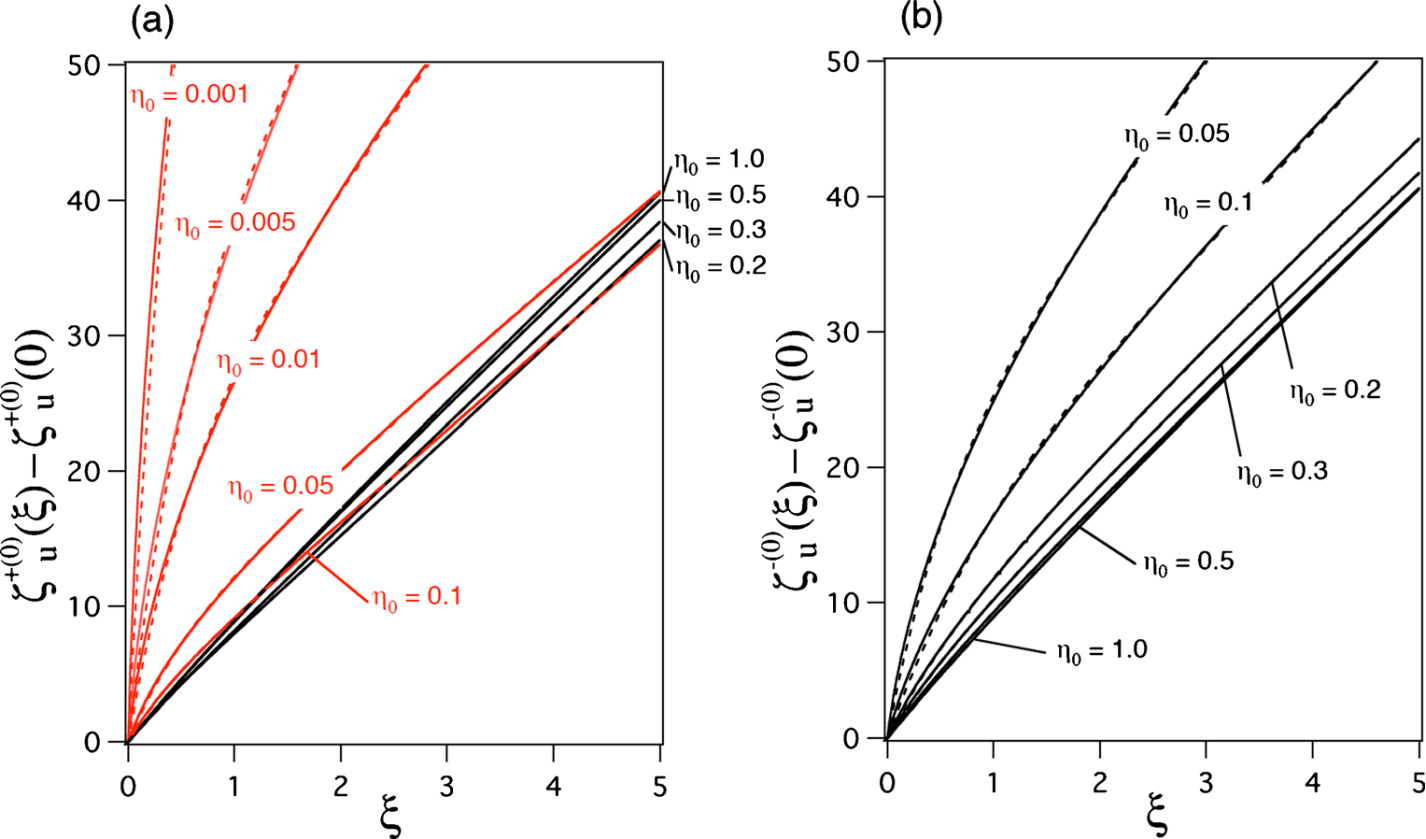
Numerical calculations of the even (+) and odd (−) zeroth-order unified separation quantities, *ζ*_*u*_^+ (0)^(*ξ*) and *ζ*_*u*_^− (0)^(*ξ*), as functions of *ξ* for various values of *η*_0_(=*ρ*_0_/*λ*_0_) when *ε*_*d*_ = 1 and *ε*_*m*_ = − 20 are used in (61) and (62), respectively. **a** Shows *ζ*_*u*_^+ (0)^(*ξ*) − *ζ*_*u*_^+ (0)^(0) with *red and black solid lines* for 0.001 ≤ *η*_0_ ≤ 0.1 and 0.2 ≤ *η*_0_ ≤ 1.0, respectively, while **b** shows *ζ*_*u*_^− (0)^(*ξ*) − *ζ*_*u*_^− (0)^(0) with a *black solid line*. The broken lines are fitting curves based on (63)

Apparently, it is very difficult to analytically solve (61) and (62) for *ζ*_*u*_^+ (0)^(*ξ*) and *ζ*_*u*_^− (0)^(*ξ*), respectively. In this case, we choose to solve them numerically. In numerical calculations, we use the dielectric permittivity *ε*_*d*_ = 1 and the metallic permittivity *ε*_*m*_ = −20 by assuming that the dielectric matter is air with a permittivity of *ε*_*d*_ = 1 and the metallic matter is gold with a permittivity of *ε*_*m*_ = − 20.6 + 1.57*i* at a wavelength of 750 nm [45]; the imaginary part of *ε*_*m*_ is significantly smaller than the real part and thus can be ignored for the sake of simplicity. For the numerical calculations, we used a set of Fortran subroutines provided by Gil et al. [46] for computing *U*(*a*, *x*) to numerically solve characteristic Eqs. (61) and (62) in *Mathematica* (Wolfram Research, Inc.). Without this set, we were not able to obtain the whole profiles of Figs. 3 and 4 from (61) and (62) by employing the usual methods of defining *U*(*a*, *x*) as in *Mathematica*. The elementary outline of how to call a Fortran subroutine from *Mathematica* can be found in [47].

Figure 3 shows *ζ*_*u*_^+ (0)^(0) and *ζ*_*u*_^− (0)^(0) as functions of *η*_0_, which are obtained by numerically solving (61) and (62), respectively, under the conditions *ξ* = 0, *ε*_*d*_ = 1, and *ε*_*m*_ = − 20. From the two profiles in Fig. 3, we certainly infer that *ζ*_*u*_^+ (0)^(0) and *ζ*_*u*_^− (0)^(0) approach positive and negative infinites, respectively, for *η*_0_ → 0 +; they become more equal in value with increasing *η*_0_ and they approach the same value for *η*_0_ → ∞. Note that *ζ*_*u*_^− (0)^(0) = 0 is satisfied at *η*_0_ = 0.204785 ⋅ ⋅ ⋅, though *ζ*_*u*_^+ (0)^(0) = 0 is not satisfied for any values of *η*_0_.

In parts (a) and (b) of Fig. 4, the solid lines show *ζ*_*u*_^+ (0)^(*ξ*) − *ζ*_*u*_^+ (0)^(0) and *ζ*_*u*_^− (0)^(*ξ*) − *ζ*_*u*_^− (0)^(0), respectively, as a function of *ξ* for specific values of *η*_0_; they are obtained by numerically solving (61) and (62), respectively, under the conditions *ε*_*d*_ = 1 and *ε*_*m*_ = − 20. The broken lines in Fig. 4 are fitting curves for the respective solid lines, though they are unclear due to their overlapping with the solid lines. In Fig. 4a, the red and black lines are used for 0.001 ≤ *η*_0_ ≤ 0.1 and 0.2 ≤ *η*_0_ ≤ 1.0, respectively, because the behavior of profiles is clearly expressed. For the curve fitting, taking into account the approximate behavior of *ζ*_*u*_^*s*(0)^(*ξ*), *s* =+, − for large values of *ξ* (described in Appendix B), we select a figure-of-merit function with four parameters, *p*_0_, *p*_1_, *p*_2_, and *p*_3_, expressed as follows:

63 $$ {\zeta}_u^{s(0)}\left(\xi \right)-{\zeta}_u^{s(0)}(0)=4{\pi}^2{p}_0\xi \exp \left[-{p}_1\xi -{p}_2{\xi}^2-{p}_3{\xi}^3\right]+4{\pi}^2{\left({\varepsilon}_d^{1/2}+{\left|{\varepsilon}_m\right|}^{1/2}\right)}^{-1}{\left({\varepsilon}_d^{-3/2}-{\left|{\varepsilon}_m\right|}^{-3/2}\right)}^{-1}\xi $$

which implies

64 $$ {\left.{\zeta^{\prime}}_u^{s(0)}(0)={d}_{\xi }{\zeta}_u^{s(0)}\left(\xi \right)\right|}_{\xi =0}=\underset{\xi \to 0}{ \lim}\left\{{\zeta}_u^{s(0)}\left(\xi \right)-{\zeta}_u^{s(0)}(0)\right\}/\xi =4{\pi}^2{p}_0+4{\pi}^2{\left({\varepsilon}_d^{1/2}+{\left|{\varepsilon}_m\right|}^{1/2}\right)}^{-1}{\left({\varepsilon}_d^{-3/2}-{\left|{\varepsilon}_m\right|}^{-3/2}\right)}^{-1}. $$

In Tables 1 and 2, we show several values of *p*_0_, *p*_1_, *p*_2_, and *p*_3_ for specific values of *η*_0_ obtained by fitting (63) to the solid curves in Fig. 4a, b, respectively (to maintain clarity in the diagram, some of the curves are not shown).

**Table 1:** Values of the parameters of the even (+) symmetry in (63) for the specific value of *η*_0_(=*ρ*_0_/*λ*_0_) obtained by fitting the solid curves in Fig. 4a

*η*_0_	*ξ*_*u*_^+ (0)^(0)	*p*_0_	*p*_1_	*p*_2_	*p*_3_
0.001	18.84	3.612	0.7220	−0.1511	0.01279
0.005	8.15	1.468	0.7605	−0.1544	0.01305
0.01	6.29	0.9580	0.7937	−0.1571	0.01329
0.05	4.84	0.2872	1.002	−0.1726	0.01543
0.1	5.40	0.1453	1.332	−0.2397	0.03138
0.2	7.42	0.05859	1.411	−0.2475	0.01687
0.3	10.16	0.04597	0.9574	−0.3140	0.04744
0.4	13.56	0.05125	0.4938	−0.08036	0.00611
0.5	17.49	0.05940	0.4538	−0.06746	0.004894
0.6	21.76	0.06257	0.4314	−0.06117	0.004304
0.7	26.22	0.06254	0.4119	−0.05723	0.004006
0.8	30.78	0.06079	0.3867	−0.05144	0.003502
0.9	35.41	0.05861	0.3662	−0.044767	0.003228
1.0	40.08	0.05641	0.3458	−0.04332	0.002860

The values of *ζ*_*u*_^+ (0)^(0) are numerically calculated in the same manner as that employed for drawing the thick line in Fig. 3

**Table 2:** Values of the parameters of the odd (−) symmetry in (63) for the specific value of *η*_0_(=*ρ*_0_/*λ*_0_) obtained by fitting the curves in Fig. 4(b)

*η*_0_	*ξ*_*u*_^+ (0)^(0)	*p*_0_	*p*_1_	*p*_2_	*p*_3_
0.05	−43.16	0.8582	0.7750	−0.1542	0.01294
0.1	−15.71	0.4228	0.7070	−0.1287	0.01043
0.2	−0.40	0.2020	0.6735	−0.1129	0.008751
0.204785	0	0.1975	0.6733	−0.1126	0.008711
0.3	6.71	0.1287	0.6381	−0.1039	0.007844
0.4	12.07	0.09537	0.5768	−0.09116	0.006756
0.5	16.88	0.07936	0.5162	−0.07867	0.005717
0.6	21.52	0.07075	0.4644	−0.06787	0.004841
0.7	26.13	0.06553	0.4234	−0.05911	0.004121
0.8	30.75	0.06181	0.3903	−0.05191	0.003519
0.9	35.40	0.05910	0.3682	−0.04778	0.003212
1.0	40.07	0.05656	0.3466	−0.04346	0.002870

The values of *ζ*_*u*_^− (0)^(0) are numerically calculated in the same manner as that employed for drawing the thin line in Fig. 3

### Unified Radial Functions of the Zeroth-Order for the Parabolic Metal Groove

In the parabolic metal groove, the zeroth-order unified separation quantities *ζ*_*u*_^+ (0)^(*ξ*) and *ζ*_*u*_^− (0)^(*ξ*) determined by (61) and (62), respectively, are quite accurately estimated by the figure-of-merit function in (63). Even with it, we cannot solve the zeroth-order unified radial Eq. (47) as an already-known differential equation. In this subsection, we demonstrate that (47) roughly approximates the modified parabolic cylinder Eq. (A.7) through transformations.

By defining the modified wave numbers of SPPs in the planar geometry, *k*_*mp*_^*s*^(*ξ*) for *s* =+, −, as

65 $$ {k}_{mp}^s\left(\xi \right)={\lambda_0}^{-1}\sqrt{\lambda_0^2{k}_p^2+\left\{{\zeta}_u^{s(0)}\left(\xi \right)-{\zeta}_u^{s(0)}(0)\right\}/\xi }, $$

we can express the zeroth-order unified radial Eq. (47) as

66 $$ {\partial}_{\sqrt{\xi}\sqrt{\xi }}{\varXi}_u^{s(0)}\left(\xi \right)+\left[{\lambda}_0^2{\left[{k}_{mp}^s\left(\xi \right)\right]}^2{\left(\sqrt{\xi}\right)}^2+{\zeta}_u^{s(0)}(0)\right]{\varXi}_u^{s(0)}\left(\xi \right)=0, $$

which can be transformed into the modified parabolic cylinder Eq. (A.7) if *k*_*mp*_(*ξ*) is constant. We solve (66) under the rough approximation

67 $$ {k}_{mp}^s\left(\xi \right)\approx {k}_{mp}^s(0), $$

in which (66) approximate

68 $$ {\partial}_{\sqrt{\xi}\sqrt{\xi }}{\varXi}_u^{s(0)}\left(\xi \right)+\left[{\lambda}_0^2{\left[{k}_{mp}^s(0)\right]}^2{\left(\sqrt{\xi}\right)}^2+{\zeta}_u^{s(0)}(0)\right]{\varXi}_u^{s(0)}\left(\xi \right)=0 $$

where

69 $$ {k}_{mp}^s(0)={\lambda_0}^{-1}\sqrt{\lambda_0^2{k}_p^2+{\zeta^{\prime}}_u^{s(0)}(0)} $$

from (65). The rough approximation in (67) can be numerically estimated by the following ratio:

70 $$ \frac{k_{mp}^s\left(\xi \right)}{k_{mp}^s(0)}=\sqrt{1+{p}_0\frac{ \exp \left[-{p}_1\xi -{p}_2{\xi}^2-{p}_3{\xi}^3\right]-1}{-{\varepsilon}_d\left|{\varepsilon}_m\right|{\left({\varepsilon}_d-\left|{\varepsilon}_m\right|\right)}^{-1}+{p}_0+{\left({\varepsilon}_d^{1/2}+{\left|{\varepsilon}_m\right|}^{1/2}\right)}^{-1}{\left({\varepsilon}_d^{-3/2}-{\left|{\varepsilon}_m\right|}^{-3/2}\right)}^{-1}}}, $$

which can be obtained from (63), (64), (65), and (69). By using the values of *p*_0_, *p*_1_, *p*_2_, and *p*_3_ in Tables 1 and 2 to numerically estimate (70), we can make graphs of the ratio *k*_*mp*_^+^(*ξ*)/*k*_*mp*_^+^(0) and *k*_*mp*_^−^(*ξ*)/*k*_*mp*_^−^(0), respectively; they are shown in parts (a) and (b) of Fig. 5, respectively. It is shown in both parts (a) and (b) of Fig. 5 that (67) is better satisfied with an increasing value of *η*_0_ and with a decreasing value of *ξ* to zero. Numerically, (67) is reasonably satisfied at *ξ* = 5 for *η*_0_ = 1.0, 0.5, 0.3, 0.2, 0.1, and 0.05 in Fig. 5a and for *η*_0_ = 1.0, 0.5, 0.3, 0.2, and 0.1 in Fig. 5b; (67) is barely satisfied at *ξ* = 5 for *η*_0_ = 0.01 in Fig. 5a and for *η*_0_ = 0.05 in Fig. 5b; (67) is scarcely satisfied at *ξ* = 5 for *η*_0_ = 0.005 and 0.001 in Fig. 5a.

**Fig. 5:**
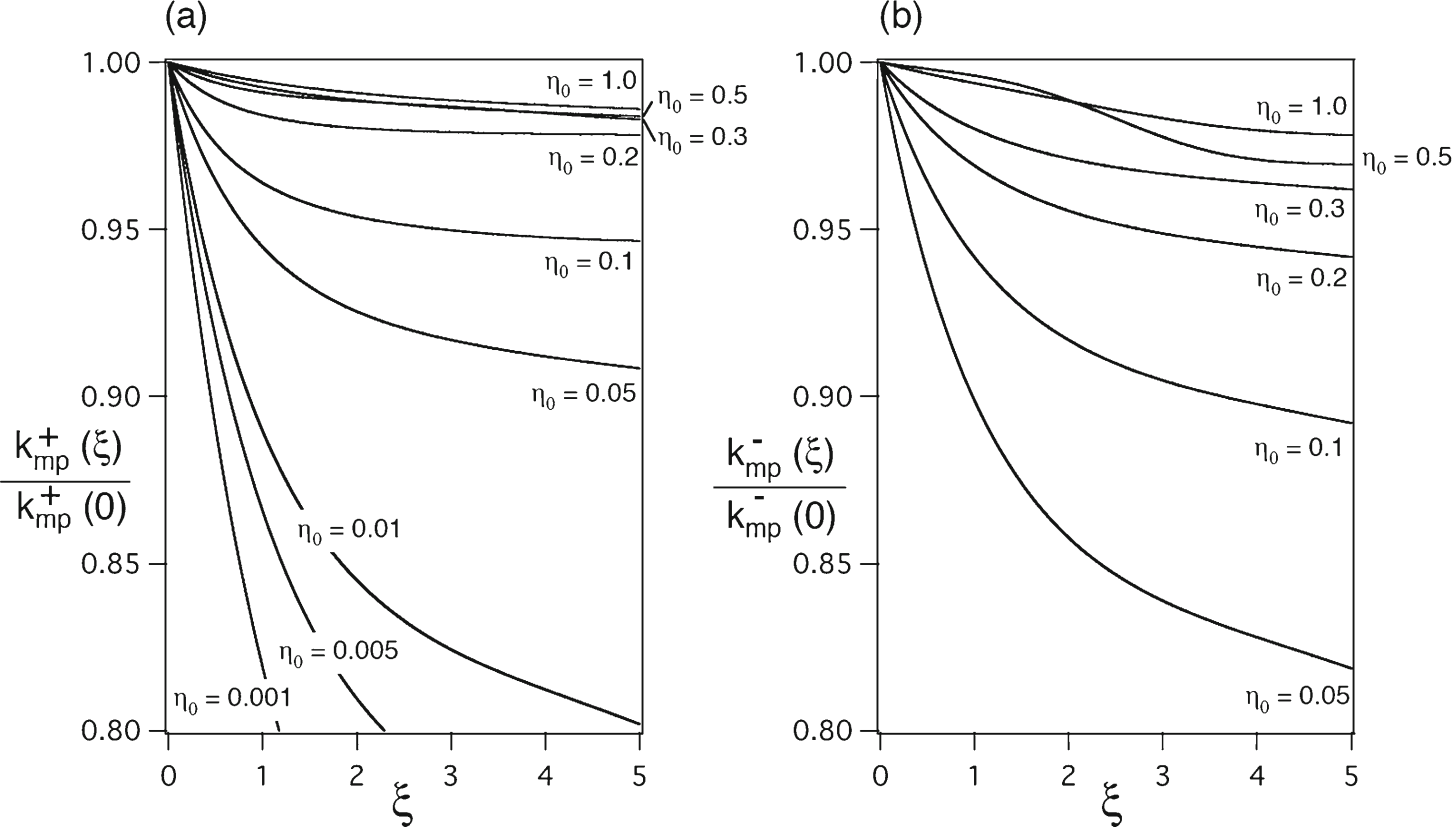
Numerical calculations of the ratio *k*_*mp*_^*s*^(*ξ*)/*k*_*mp*_^*s*^(0) in (70) for **a** *s* = + and **b** *s* = − as a function of *ξ* for various values of *η*_0_(=*ρ*_0_/*λ*_0_) obtained by using *ε*_*d*_ = 1, *ε*_*m*_ = − 20, and specific values of *p*_0_, *p*_1_, *p*_2_, and *p*_3_ in Tables 1 and 2

The approximate differential Eq. (68) of the zeroth-order unified radial equation is a special case of the modified parabolic cylinder Eq. (A.7) corresponding to the parameter values

71 $$ z=\sqrt{2{\lambda}_0{k}_{mp}^s(0)\xi}\equiv {u}_r^s\left(\xi \right),a=-{\zeta}_u^{s(0)}(0)/2{\lambda}_0{k}_{mp}^s(0)\equiv {v}_r^s. $$

Because of (A.17), outgoing and incoming solutions for (A.7) are given by *E*(*a*, *z*) and E*(*a*, *z*), respectively; by taking (48) and (50) into account, we can choose

72 $$ \begin{array}{c}\hfill {\varXi}_{out}^{s(0)}\left(\xi \right)={H}_0^{\prime }E\left({v}_r^s,{u}_r^s\left(\xi \right)\right) \exp \left(\mathit{\hbox{--}}i\varphi \right)\hfill \\ {}\hfill {\varXi}_{in}^{s(0)}\left(\xi \right)={H}_0^{\prime }{E}^{*}\left({v}_r^s,{u}_r^s\left(\xi \right)\right) \exp \left(i\varphi \right)\hfill \end{array} $$

where

73 $$ \varphi ={ \tan}^{-1}\left[\sqrt{1+ \exp \left(2\pi {v}_r^s\right)}- \exp \left(\pi {v}_r^s\right)\right] $$

74 $$ {H}_0^{\prime }={2}^{1/4}{H}_0{\left[1+ \exp \left(2\pi {v}_r^s\right)\right]}^{-1/4}\frac{\sqrt{\left|\varGamma \left(3/4+i{v}_r^s/2\right)\right|}}{\sqrt{\left|\varGamma \left(1/4+i{v}_r^s/2\right)\right|}} $$

are obtained by using (A.9), (A.10), (A.16), and (A.17). For the limit of *ξ* → ∞, from the asymptotic formulae (A.17) and (A.18), it is inferred that

75 $$ {\varXi}_{out}^{s(0)}\left(\xi \right)\sim {H}_0^{\prime}\sqrt{2/{u}_r^s\left(\xi \right)}{e}^{i{\alpha}^s\left(\xi \right)},{\varXi}_{in}^{s(0)}\left(\xi \right)\sim {H}_0^{\prime}\sqrt{2/{u}_r^s\left(\xi \right)}{e}^{-i{\alpha}^s\left(\xi \right)}\left(\xi \to \infty \right) $$

where

76 $$ {\alpha}^s\left(\xi \right)={4}^{-1}{\left\{{u}_r^s\left(\xi \right)\right\}}^2-{v}_r \ln {u}_r^s\left(\xi \right)+{2}^{-1} \arg \varGamma \left({2}^{-1}+i{v}_r^s\right)+\pi /4-\varphi . $$

By using (49) for (72), we obtain

77 $$ {\varXi}_u^{s(0)}\left(\xi \right)=2{H}_0^{\prime}\left[\sqrt{\mathrm{co}{\mathrm{s}}^3\varphi / \sin \varphi }W\left({v}_r^s,{u}_r^s\left(\xi \right)\right)+\sqrt{{ \sin}^3\varphi / \cos \varphi }W\left({v}_r^s,-{u}_r^s\left(\xi \right)\right)\right], $$

which indicates neither outgoing nor incoming waves but a standing wave with the free end. The qualitative behavior of (77) as a function of *ξ* can be understood from properties of *W*(*a*, *z*) and *W*(*a*, − *z*) as two linearly independent solutions to the differential Eq. (A.7). As described in 3.3 [38], when *a* is negative, (A.7) has oscillatory-type solutions throughout; when *a* is positive, (A.7) has exponential-type solutions for $-2\sqrt{a}<z<2\sqrt{a}$ and oscillatory-type solutions for $-\infty <z<-2\sqrt{a}$ and $2\sqrt{a}<z<+\infty$. Because it follows from (71) that *a* = − *ζ*_*u*_^*s*(0)^(0)/2*λ*_0_ *k*_*mp*_^*s*^(0) is given for *W*(*a*, *z*) and *W*(*a*, − *z*) in (77), the qualitative behavior of *Ξ*_*u*_^*s*(0)^(*ξ*) in (77) can be determined by the positive and negative values of *ζ*_*u*_^*s*(0)^(0): *Ξ*_*u*_^*s*(0)^(*ξ*) is oscillatory as a function of *ξ* ∈ [0, ∞) for *ζ*_*u*_^*s*(0)^(0) > 0, while *Ξ*_*u*_^*s*(0)^(*ξ*) changes from exponential to oscillatory behaviors with an increasing value of *ξ* ∈ [0, ∞) for *ζ*_*u*_^*s*(0)^(0) < 0. For *ζ*_*u*_^+ (0)^(0) in Fig. 3, because of *ζ*_*u*_^+ (0)^(0) > 0 for all the values of *η*_0_, *Ξ*_*u*_^+ (0)^(*ξ*) is oscillatory as a function of *ξ* ∈ [0, ∞) for any values of the radius of curvature, *ρ*_0_. For *ζ*_*u*_^− (0)^(0) in Fig. 3, *Ξ*_*u*_^− (0)^(*ξ*) is oscillatory when *ζ*_*u*_^− (0)^(0) > 0 (i.e., *η*_0_ > 0.204 ⋅ ⋅ ⋅), while *Ξ*_*u*_^− (0)^(*ξ*) changes from exponential to oscillatory behaviors with an increasing value of *ξ* ∈ [0, ∞) when *ζ*_*u*_^− (0)^(0) < 0 (i.e., 0 < *η*_0_ < 0.204 ⋅ ⋅ ⋅).

## Non-Existence of Zeroth-Order Solutions for the Parabolic Metal Wedge

In this section, we clearly prove that no plasmonic modes exist for the parabolic metal wedge by examining (43).

For the parabolic metal wedge, we can write *ε*_1_ = *ε*_*m*_ < 0 and *ε*_2_ = *ε*_*d*_ > 0. Substituting *ε*_2_ = *ε*_*d*_ into (43) for *j* = 2, we have

78 $$ {\partial}_{\sqrt{\eta}\sqrt{\eta }}{H}_2^{s(0)}\left(\sqrt{\eta },\xi \right)+\left[-{\zeta}_u^{s(0)}\left(\xi \right)-{\lambda}_0^2{\beta}_d^2{\left(\sqrt{\xi}\right)}^2+4{\pi}^2{\varepsilon}_d{\left(\sqrt{\eta}\right)}^2\right]{H}_2^{s(0)}\left(\sqrt{\eta },\xi \right)=0, $$

which is the same as (52) by replacing ${H}_2^{s(0)}\left(\sqrt{\eta },\xi \right)$ with ${H}_1^{s(0)}\left(\sqrt{\eta },\xi \right)$ in (78) and therefore can be solved in the same manner as that employed in (52). Dividing (78) by 4*πε*_*d*_^1/2^ and rearranging terms, we obtain the same Eq. as (53), a special case of the modified parabolic cylinder Eq. (A.7) corresponding to the parameter values given by (54). Since two standard linearly independent solutions to (A.7) are given by *W*(*a*, *z*) and *W*(*a*, − *z*) written in (A.8), we observe that two linearly independent solutions to (78) are expressed by

79 $$ {H}_2^{s(0)}\left(\sqrt{\eta },\xi \right)=W\left({v}_d^s\left(\xi \right),{u}_d\left(\sqrt{\eta}\right)\right),W\left({v}_d^s\left(\xi \right),-{u}_d\left(\sqrt{\eta}\right)\right). $$

By using (A.19), we find that the asymptotic behaviors of the two solutions in (79) are oscillatory as a function of *η*, with the amplitude varying as *η* ^− 1/4^ for the limit of $\sqrt{\eta}\to \pm \infty$; this behavior is similar to those exhibited by the outgoing and incoming solutions of the unified radial equation for *ξ* → ∞ in (75), respectively. This clearly indicates that the two linearly independent solutions in (79) are unsuitable for the composition of the zeroth-order extended angular function in the interval $\pm \sqrt{\eta}\in \left[\sqrt{\eta_0},\infty \right)$ because their behavior does not belong to localization but to propagation. Therefore, we can arrive at the very interesting conclusion that plasmonic modes do not exist for the parabolic metal wedge.

## Electric Field-Line Representation

It is very important to understand plasmonic modes not only mathematically but also visually. In the present case where the electromagnetic field has only a *z*-component of the magnetic field in the parabolic cylinder coordinate system, we can use a field-line pattern of the electric field, which is graphically simpler and more intuitive than the field-vector pattern representation.

We describe the field-line pattern representation of an electric field in detail. The tangent at an arbitrary point on an electric field line indicates the direction of electric field vector $E\left(\xi, \sqrt{\eta },t\right)$ at this point. This can be described mathematically by using a line vector element *d* ***s*** as $E\left(\xi, \sqrt{\eta },t\right)\times ds=\mathbf{0}$, which can be simplified into the form

80 $$ \mathrm{d}\xi /{E}_{\xi}\left(\xi, \sqrt{\eta },t\right)\sqrt{\xi }-d\eta /{E}_{\eta}\left(\xi, \sqrt{\eta },t\right)\sqrt{\eta }=0 $$

when $E\left(\xi, \sqrt{\eta },t\right)={E}_{\xi}\left(\xi, \sqrt{\eta },t\right){e}_{\xi }+{E}_{\eta}\left(\xi, \sqrt{\eta },t\right){e}_{\eta }$ and *ds* = *h*_*ξ*_d*ξe*_*ξ*_ + *h*_*η*_ *dηe*_*η*_, where ***e***_*ξ*_ and ***e***_*η*_ are the unit vectors along the *ξ*- and *η*-axes, respectively; *h*_*ξ*_ and *h*_*η*_ denote the metric coefficients of the parabolic cylindrical coordinates in (1) given by ${h}_{\xi }={2}^{-1}{\lambda}_0\sqrt{\left(\xi +\eta \right)/\xi }$ and ${h}_{\eta }={2}^{-1}{\lambda}_0\sqrt{\left(\xi +\eta \right)/\eta }$. By using (31)–(33) for (80), we obtain a differential equation expressed in terms of the total derivatives with respect to *ξ* and *η* as follows:

81 $$ {\partial}_{\xi }{H}_z\left(\xi, \sqrt{\eta },t-\pi /2\omega \right)d\xi +{\partial}_{\eta }{H}_z\left(\xi, \sqrt{\eta },t-\pi /2\omega \right)d\eta =0, $$

which becomes the exact differential equation *dψ*(*ξ*, *η*) = 0 with the solution *ψ*(*ξ*, *η*) = constant, if we set $\psi \left(\xi, \eta \right)={H}_z\left(\xi, \sqrt{\eta },t-\pi /2\omega \right)$. Then, for the time-varying scalar field, *f*(*ξ*, *η*, *t*), of the electric field-line representation, we can set

82 $$ f\left(\xi, \eta, t\right)={H}_z\left(\xi, \sqrt{\eta },t-\pi /2\omega \right). $$

The field-line pattern at time *t* = *t*_0_ is described by the scalar field with the contour *f*(*ξ*, *η*, *t*_0_) = *C*, where *C* indicates the contour level. The contour interval can be controlled by the interval value of the contour level. The evolution of field-line patterns can be investigated for different moments: *t* = *t*_0_ + *nτ* with *n* = 1, 2, 3, ⋅ ⋅ ⋅, where *τ* denotes a suitable chosen duration between two neighboring snapshots.

For actual calculations, using (4), (7), and (25), we rewrite (82) in a form that contains the unified radial function and the extended angular function with even (+) and odd (−) symmetries. The scalar field of the zeroth-order approximation for (82) is given by

83 $$ {f}^{s(0th)}\left(\xi, \eta, t\right)=\mathrm{R}\mathrm{e}\left[{\varXi}_u^{s(0)}\left(\xi \right){H}_j^{s(0)}\left(\sqrt{\eta },\xi \right) \exp \left(-i\omega t+i\pi /2\right)\right],j=1,2fors=+,-. $$

**Fig. 6:**
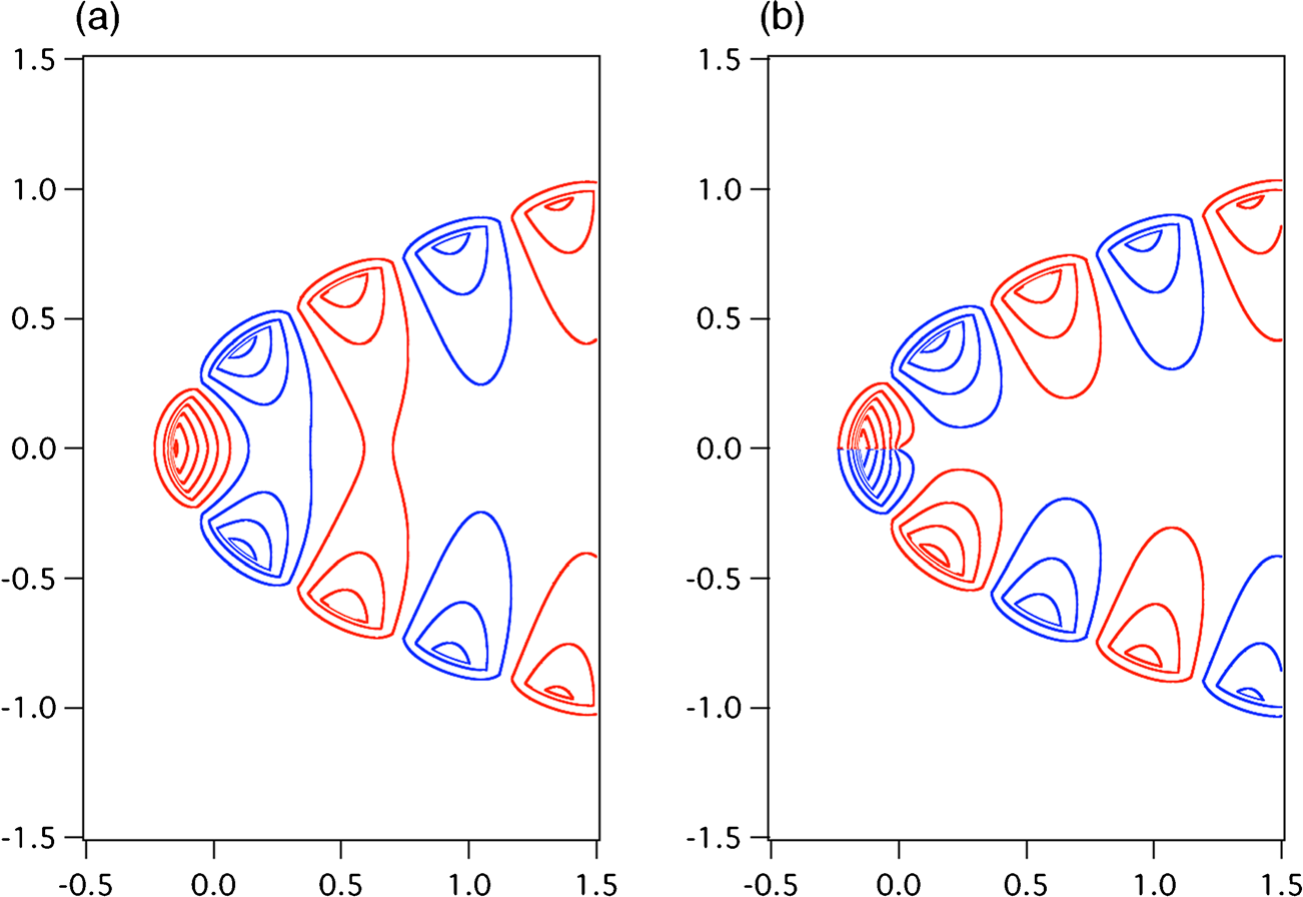
Electric field lines of the zeroth-order plasmonic mode of the **a** even and **b** odd symmetries in the parabolic metal groove for the surface *η*_0_(=*ρ*_0_/*λ*_0_) = 0.3, obtained using (83) at *t* = *π*/2*ω* under the conditions *ε*_*d*_ = 1 and *ε*_*m*_ = − 20. The *blue and red lines* indicate the clockwise and counterclockwise loops, respectively, of the line of electric force. The geometric dimensions of the horizontal and vertical axes are normalized by the wavelength in vacuum *λ*_0_

**Fig. 7:**
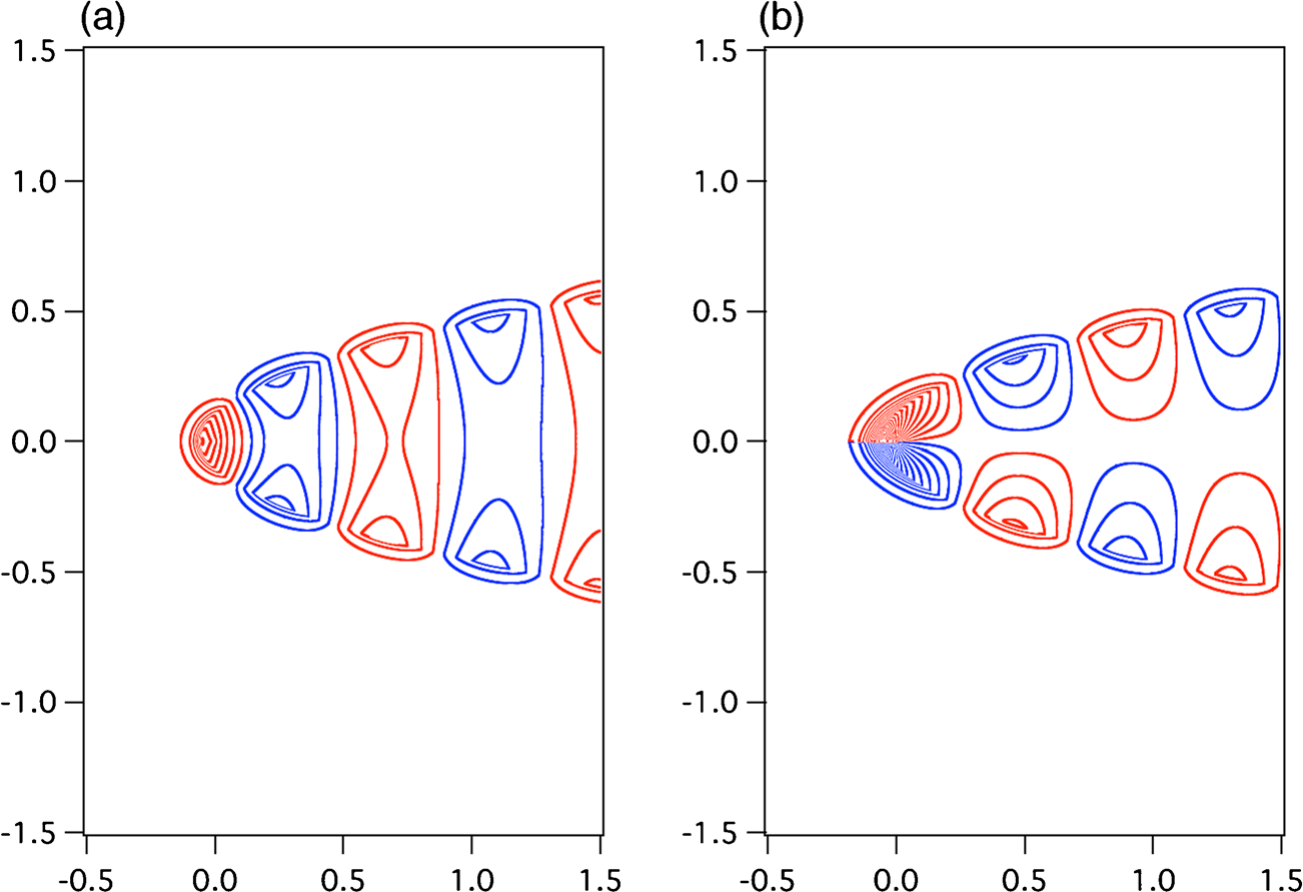
Electric field lines of the zeroth-order plasmonic mode of the **a** even and **b** odd symmetries in the parabolic metal groove for the surface *η*_0_(=*ρ*_0_/*λ*_0_) = 0.1, obtained using (83) at *t* = *π*/2*ω* under the conditions *ε*_*d*_ = 1 and *ε*_*m*_ = − 20. The *blue and red lines* indicate the clockwise and counterclockwise loops, respectively, of the line of electric force. The geometric dimensions of the horizontal and vertical axes are normalized by the wavelength in vacuum *λ*_0_

Figures 6 and 7 show the electric field-line patterns of the zeroth-order approximated plasmonic modes of (a) even and (b) odd symmetries in the parabolic metal groove for the surfaces *η*_0_ = 0.3 and *η*_0_ = 0.1, respectively, where the surface *η*_0_ is characterized in (2). The electric field-line patterns were obtained by using (83) with *t* = *π*/2*ω*. In the calculations, for the even symmetry, we employed ${H}_1^{+(0)}\left(\sqrt{\eta },\xi \right)$, ${H}_2^{+(0)}\left(\sqrt{\eta },\xi \right)$, and *Ξ*_*u*_^+ (0)^(*ξ*) in (55), (60), and (77), respectively; for the odd symmetry, we used ${H}_1^{-(0)}\left(\sqrt{\eta },\xi \right)$, ${H}_2^{-(0)}\left(\sqrt{\eta },\xi \right)$, and *Ξ*_*u*_^− (0)^(*ξ*) in (56), (60), and (77), respectively. The parameters *ε*_*d*_ = 1, *ε*_*m*_ = − 20, and *H*_0_^′^ = 1 were used. Some specific values for *ζ*_*u*_^+ (0)^(*ξ*) and *ζ*_*u*_^− (0)^(*ξ*) are given in Tables 1 and 2, respectively. The geometric dimensions in Figs. 6 and 7 are normalized by the wavelength in vacuum, *λ*_0_, on the horizontal and vertical axes. The blue and red lines in Figs. 6 and 7 indicate clockwise and counterclockwise loops, respectively, for the line of electric force. The field patterns in Figs. 6 and 7 evolve with the behavior of the standing waves. By comparing Figs. 6 and 7, we can consider a strong contrast between even and odd symmetries for the electric field-line patterns in parabolic metal grooves. For parts (b) of Figs. 6 and 7, the apparent distinction can be found in the behavior of the electric field-line patterns around the origin of figures: the electric field-lines are more densely distributed around the origin of figures for Fig. 7b than around that for Fig. 6b. This distinction is not observed for parts (a) of Figs. 6 and 7. The clear contrast of even and odd symmetries for the electric field-line patterns can be understood from the viewpoint of the positive and negative values of *ζ*_*u*_^*s*(0)^(0) for *s* =+, −, by observing that *ζ*_*u*_^− (0)^(0) = 6.71 > 0 and *ζ*_*u*_^− (0)^(0) = − 15.71 < 0 are obtained for parts (b) of Figs. 6 and 7, respectively, while *ζ*_*u*_^+ (0)^(0) = 10.16 > 0 and *ζ*_*u*_^+ (0)^(0) = 5.40 > 0 are for parts (a) of Figs. 6 and 7, respectively (these data given in Tables 1 and 2). We can observe that the dense distribution of electric field lines around the origin of figures is caused by the negative value of *ζ*_*u*_^*s*(0)^(0), because, from (77) with (73), *Ξ*_*u*_^*s*(0)^(0) largely depends on *ζ*_*u*_^*s*(0)^(0):

84 $$ {\varXi}_u^{s(0)}(0)=2{H}_0^{\prime}\left[\sqrt{\mathrm{co}{\mathrm{s}}^3\varphi / \sin \varphi }+\sqrt{{ \sin}^3\varphi / \cos \varphi}\right]W\left(-{\zeta}_u^{s(0)}(0)/2{\gamma}^s,0\right), $$

where *γ* ^*s*^ = *λ*_0_ *k*_*mp*_^*s*^(0); (84) can be transformed for *ζ*_*u*_^*s*(0)^(0) ≪ 0 into the form

85 $$ {\varXi}_u^{s(0)}(0)\sim 2{H}^{\prime }{\varphi}^{-1/2}W\left(-{\zeta}_u^{s(0)}(0)/2{\gamma}^s,0\right),\varphi \sim {2}^{-1} \exp \left(\pi {\zeta}_u^{s(0)}(0)/2{\gamma}^s\right) $$

which shows that *Ξ*_*u*_^*s*(0)^(0) exponentially increases with a decreasing value of *ζ*_*u*_^*s*(0)^(0) < 0 because *Ξ*_*u*_^*s*(0)^(0) is approximately described as an exponential function of − *ζ*_*u*_^*s*(0)^(0) for *ζ*_*u*_^*s*(0)^(0) ≪ 0. This qualitatively explains the numerical results: *Ξ*_*u*_^− (0)^(0) = 2.562, *Ξ*_*u*_^− (0)^(0) = 9.587, *Ξ*_*u*_^+ (0)^(0) = 2.282, *Ξ*_*u*_^+ (0)^(0) = 2.691 for *ζ*_*u*_^− (0)^(0) = 6.71 > 0, *ζ*_*u*_^− (0)^(0) = − 15.71 < 0, *ζ*_*u*_^+ (0)^(0) = 10.16 > 0, and *ζ*_*u*_^+ (0)^(0) = 5.40 > 0, respectively; they were calculated by using (84) with (73), (A.10) and *H*_0_^′^ = 1. It is important to point out that the dense distribution of electric field lines around the origin of figures takes place only for the odd symmetry because the negative value of *ζ*_*u*_^*s*(0)^(0) is limited to the odd symmetry (*s* = −) from the numerical results of *ζ*_*u*_^*s*(0)^(0) for *s* =+, − in Fig. 3.

**Fig. 8:**
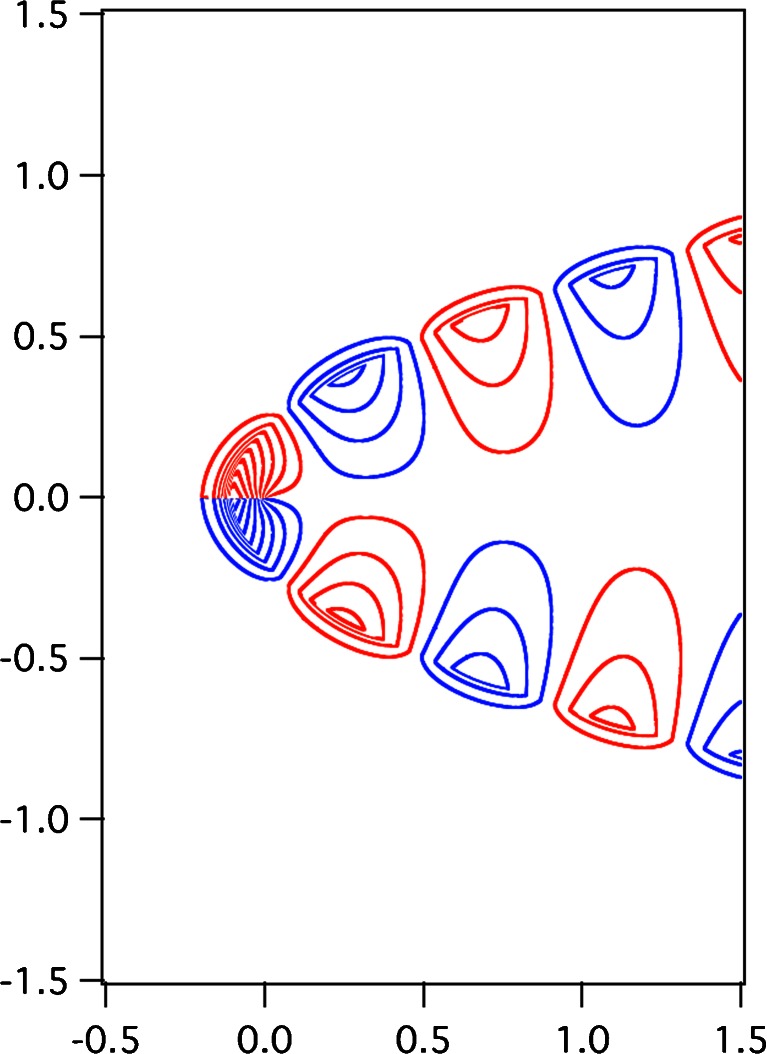
Electric field lines of the zeroth-order plasmonic mode of the odd symmetry in the parabolic metal groove for the surface *η*_0_(=*ρ*_0_/*λ*_0_) = 0.204785, the specific value of *η*_0_ for *ζ*_*u*_^− (0)^(0) = 0 in (62) when *ε*_*d*_ = 1 and *ε*_*m*_ = − 20. The other calculation conditions are the same as those in part b of Figs. 6 and 7. The *blue and red lines* indicate the clockwise and counterclockwise loops, respectively, of the line of electric force. The geometric dimensions of the horizontal and vertical axes are normalized by the wavelength in vacuum *λ*_0_

Figure 8 shows the electric field-line patterns of the zeroth-order approximated plasmonic modes of odd symmetry in the parabolic metal groove for the surface *η*_0_ = 0.204785; this is the specific value of *η*_0_ for *ζ*_*u*_^− (0)^(0) = 0 given in Table 2. The other calculation conditions for drawing Fig. 8 are identical to those in Figs. 6 and 7. By substituting *ζ*_*u*_^− (0)^(0) = 0 into (77), we simplify the unified radial function of the zeroth order into the form

86 $$ {\varXi}_u^{-(0)}\left(\xi \right)={H}_0^{\prime}\sqrt{\pi }{\left[{\gamma}^{-}\xi \right]}^{1/4}\left[{2}^{3/4}{J}_{-1/4}\left({\gamma}^{-}\xi /2\right)+{2}^{1/4}{J}_{1/4}\left({\gamma}^{-}\xi /2\right)\right],{\gamma}^{-}={\lambda}_0{k}_{mp}^{-}(0) $$

where the *J*-function is the Bessel function of the first kind; we use the formula (A.11) for $W\left(0,\pm \sqrt{2{\gamma}^{-}\xi}\right)$ and we use the specific relation, $\tan \varphi =\sqrt{2}-1$, obtained by substituting *ζ*_*u*_^− (0)^(0) = 0 into (73). In the case of *ξ* → 0, (86) becomes

87 $$ {\varXi}_u^{-(0)}(0)=2{H}_0, $$

where we use the asymptotic behavior of the Bessel function of the first kind, *J*_*ν*_(*z*) ∼ (*z*/2)^*ν*^/*Γ*(1 + *ν*) as *z* → 0 [36], and we use the relation ${H}_0^{\hbox{'}}={H}_0\sqrt{\varGamma \left(3/4\right)/\varGamma \left(1/4\right)}$, obtained by substituting *ζ*_*u*_^− (0)^(0) = 0 into (74) for *s* = −. By substituting *ζ*_*u*_^− (0)^(0) = 0 into (56) for *ξ* = 0, and by using (A.13), we obtain the angular function in the dielectric region as follows:

88 $$ {H}_1^{-(0)}\left(\sqrt{\eta },0\right)=\pm {\left(\eta /{\eta}_0\right)}^{1/4}{J}_{1/4}\left.false(\pi {\varepsilon}_d^{1/2}\eta \right)/{J}_{1/4}\left(\pi {\varepsilon}_d^{1/2}{\eta}_0\right),\pm \sqrt{\eta}\in \left[0,\sqrt{\eta_0}\right] $$

(double-sign corresponds). By substituting *ζ*_*u*_^− (0)^(0) = 0 into (60) for *s* = − and *ξ* = 0, by using (A.4), and by taking into account the odd symmetric relation in (9), we obtain the angular function in the metallic region as follows:

89 $$ {H}_2^{-(0)}\left(\sqrt{\eta },0\right)=\pm {\left(\eta /{\eta}_0\right)}^{1/4}{K}_{1/4}\left(\pi {\left|{\varepsilon}_m\right|}^{1/2}\eta \right)/{K}_{1/4}\left(\pi {\left|{\varepsilon}_m\right|}^{1/2}{\eta}_0\right),\pm \sqrt{\eta}\in \left[\sqrt{\eta_0},\infty \right) $$

(double-sign corresponds), where the *K*-function is the modified Bessel function of the second kind. Because Eqs. (86) to (89) are described by relatively simple functions such as Bessel functions, we can carefully study the electric field-line pattern of Fig. 8, calculated by using (83) with *t* = *π*/2*ω* for *s* = −, as compared to those of parts (b) of Figs. 6 and 7. For *ξ* → 0 and *t* = *π*/2*ω*, the zeroth-order scalar field in (83) for *s* = − is described as

90 $$ {f}^{-(0th)}\left(0,\eta, \pi /2\omega \right)=2{H}_0{H}_j^{-(0)}\left(\sqrt{\eta },0\right),j=1,2, $$

where ${H}_1^{-(0)}\left(\sqrt{\eta },0\right)$ and ${H}_2^{-(0)}\left(\sqrt{\eta },0\right)$ are given in (88) and (89), respectively. Equation (90) shows the limiting behavior of the electric field-line pattern along the horizontal line passing through the apex of the parabolic cylinder in Fig. 8. Because the electric field-line pattern of Fig. 8 corresponds to the case *ζ*_*u*_^− (0)^(0) = 0, it is used as a fixed standard to thoroughly understand the electric field-line pattern of parts (b) in Figs. 6 and 7 with *ζ*_*u*_^− (0)^(0) > 0 and *ζ*_*u*_^− (0)^(0) < 0, respectively.

## Conclusions

We studied plasmonic modes for a parabolic cylinder geometry by solving the wave equation for the magnetic field in parabolic cylindrical coordinates through the QSOV in combination with perturbation methods. By analytically solving the zeroth-order perturbation equations for unified radial and extended angular functions, we found that plasmonic modes are absent for the parabolic metal wedge but present in separating even and odd symmetries for the parabolic metal groove in the form of standing waves and not as superfocusing waves. For the parabolic metal groove, we showed that the unified radial function of the zeroth order is described by using the *W*-function of parabolic cylinder functions for the even and odd symmetries. We also showed that the extended angular functions of the zeroth order are described by using the *U*-function of parabolic cylinder functions in the metallic region for both the even and odd symmetries, and by using the *w*_1_- and *w*_2_-functions of those in the dielectric region for the even and odd symmetries, respectively. The unified radial and extended angular functions of the first and higher order were not shown explicitly because they could not be estimated numerically due to some difficulties involved in numerical calculations. By using the zeroth-order perturbed solutions, we obtained the electric field-line patterns of the zeroth-order approximated plasmonic modes in the parabolic metal groove for relatively small and large radii of curvature at the apex of the parabolic cylinder. The electric field-line patterns in the odd symmetry showed that the electric field around the apex of the parabolic cylinder is stronger for a small curvature radius than for a large one, while those in the even symmetry showed no such an apparent distinction; these results were analytically studied from the mathematical viewpoint of the positive and negative values of the zeroth-order unified separation quantity. We firmly believe that the QSOV method employed to solve the wave equation is the optimal method with which to theoretically understand plasmonic modes in various tapered geometries.

## Appendix A. Some Properties of Parabolic Cylinder Functions

Parabolic cylinder functions (PCFs) *U*(*a*, *z*), *V*(*a*, *z*), and *W*(*a*, *z*) are associated with the parabolic cylinder in harmonic analysis; therefore, their descriptions can be found everywhere [38, 46, 48–51]. In particular, chapter 19 of Abramowitz and Stegun [48] gives a basic overview of properties of PCFs.

The two standard linearly independent solutions of the differential equation

A.1 $$ {d}^2y/d{z}^2-\left({z}^2/4+a\right)y=0 $$

are denoted by *U*(*a*, *z*) and *y*(*a*, *z*). These solutions can be described by using the simplest even and odd functions denoted by *y*_1_(*a*, *z*) and *y*_2_(*a*, *z*), respectively (see the reference [49]). Note that the pair *y*_1_(*a*, *z*) and *y*_2_(*a*, *z*) are not numerically satisfactory [52], because they have almost the same asymptotic behavior at infinity. The behavior of *U*(*a*, *z*) and *V*(*a*, *z*) is for large positive values of *z* [50]:

A.2 $$ U\left(a,z\right)\sim \exp \left(-{z}^2/4\right){z}^{-a-1/2}\left(z\to \infty \right), $$

A.3 $$ V\left(a,z\right)\sim \sqrt{2/\pi } \exp \left({z}^2/4\right){z}^{a-1/2}\left(z\to \infty \right). $$

At *a* = 0, from (19.15.9) and (19.15.13) with (19.15.2) in [48], we have

A.4 $$ U\left(0,z\right)={\pi}^{-1/2}{\left(z/2\right)}^{1/2}{K}_{1/4}\left({z}^2/4\right), $$

A.5 $$ V\left(0,z\right)={2}^{-1}{z}^{1/2}\left[{I}_{1/4}\left({z}^2/4\right)+{I}_{-1/4}\left({z}^2/4\right)\right], $$

respectively, where the *I*- and *K*-functions represent the modified Bessel functions of the first and second kinds, respectively. The behavior of *U*(*a*, *z*) is, for large positive values of a (*a* > > *z* ^2^) [38, 48]:

A.6 $$ U\left(a,z\right)\sim \sqrt{\pi }{2}^{-a/2-1/4}{\left\{\varGamma \left(3/4+a/2\right)\right\}}^{-1} \exp \left(-{a}^{1/2}z\right)\left(a\to \infty \right). $$

The differential equation

A.7 $$ {d}^2y/d{z}^2+\left({z}^2/4-a\right)y=0 $$

is a modified form of (A.1) obtained by replacing *a* by − *ia* and *z* by *ze* ^(1/4)*iπ*^. We see from [50] that *y*_1_(−i*a*, *ze* ^(1/4)*iπ*^) and *e* ^− (1/4)*iπ*^ *y*_2_(−i*a*, *ze* ^(1/4)*iπ*^), usually rewritten as *w*_1_(*a*, *z*) and *w*_2_(*a*, *z*), respectively, are even and odd solutions, respectively, to (A.7); the series involves complex quantities in which the imaginary part of the sum vanished identically [38]. The two standard linearly independent solutions of (A.7) are denoted by *W*(*a*, *z*) and *W*(*a*, − *z*); they are given by the representation [38]

A.8 $$ W\left(a,\pm z\right)={2}^{-3/4}\left(\sqrt{G_1/{G}_3}{w}_1\left(a,z\right)\mp \sqrt{2{G}_3/{G}_1}{w}_2\left(a,z\right)\right) $$

(double-sign corresponds) where

A.9 $$ {G}_1=\left|\varGamma \left(1/4+ia/2\right)\right|,{G}_3=\left|\varGamma \left(3/4+ia/2\right)\right|. $$

At *z* = 0, we have [38]

A.10 $$ W\left(a,0\right)={2}^{-3/4}\sqrt{G_1/{G}_3}. $$

At *a* = 0, we have [38, 48]

A.11 $$ W\left(0,\pm z\right)={2}^{-5/4}\sqrt{\pi z}\left[{J}_{\hbox{--} 1/4}\left({z}^2/4\right)\mp {J}_{1/4}\left({z}^2/4\right)\right], $$

(double-sign corresponds) where the *J*-function is the Bessel function of the first kind. By comparing (A.11) with (A.8) for *a* = 0, we obtain

A.12 $$ {w}_1\left(0,\pm z\right)={2}^{-1/4}\pi {\left\{\varGamma \left(1/4\right)\right\}}^{-1}\sqrt{z}{J}_{-1/4}\left({z}^2/4\right), $$

A.13 $$ {w}_2\left(0,\pm z\right)=\pm {2}^{-5/4}\varGamma \left(1/4\right)\sqrt{z}{J}_{1/4}\left({z}^2/4\right), $$

(double-sign corresponds) where we use the relation $\varGamma \left(1/4\right)\varGamma \left(3/4\right)=\sqrt{2}\pi$, obtained by substituting *z* = 1/4 into the reflection formula for the gamma function [36]: *Γ*(*z*)*Γ*(1 − *z*) = *π*/sin *πz*. The complex solutions of (A.7) are defined as [38, 48]

A.14 $$ E\left(a,z\right){k}^{-1/2}W\left(a,z\right)+i{k}^{1/2}W\left(a,-z\right) $$

A.15 $$ {E}^{*}\left(a,z\right)={k}^{-1/2}W\left(a,z\right)-i{k}^{1/2}W\left(a,-z\right) $$

where

A.16 $$ k=\sqrt{1+{e}^{2\pi a}}-{e}^{\pi a}. $$

The behaviors of *E*(*a*, *z*) and *E**(*a*, *z*) are, for large positive values of *z* [38, 48]:

A.17 $$ E\left(a,z\right)\sim \sqrt{2/z} \exp \left[i\varPhi \right],{E}^{*}\left(a,z\right)\sim \sqrt{2/z} \exp \left[-i\varPhi \right]\left(z\to \infty \right) $$

where

A.18 $$ \Phi ={z}^2/4-a \ln z+\pi /4+{\phi}_2/2,{\phi}_2= \arg \varGamma \left(1/2+ia\right). $$

Therefore, *E*(*a*, *z*) and *E**(*a*, *z*) behave as outgoing and incoming waves, respectively, for large *z*. From (A.14), (A.15), and (A.17), the behaviors of *W*(*a*, *z*) and *W*(*a*, − *z*) are, for large positive values of *z* [38, 46]:

A.19 $$ W\left(a,z\right)\sim \sqrt{\frac{2k}{z}} \cos \Phi, W\left(a,-z\right)\sim \sqrt{\frac{2}{kz}} \sin \Phi \left(z\to \infty \right) $$

From (291)–(292) in [38] or 19.22.1–19.22.2 in [48], we see that the behaviors of *W*(*a*, *z*) and *W*(*a*, − *z*) are, for large positive values of *a* (*a* > > *z* ^2^):

A.20 $$ W\left(a,\pm z\right)\sim {2}^{-3/4}\sqrt{G_3/{G}_1} \exp \left(\mp z\sqrt{a}\right)\left(a\to \infty \right) $$

(double-sign corresponds). By using (A.8) with (A.20) we find for large positive values of *a* (*a* > > *z* ^2^) that

A.21 $$ {w}_1\left(a,z\right)\sim \cosh \left(z\sqrt{a}\right),{w}_2\left(a,z\right)\sim {G}_1/\left(\sqrt{2}{G}_3\right) \sinh \left(z\sqrt{a}\right)\left(a\to \infty \right) $$

## Appendix B. Approximate Behaviors of *ζ*_*u*_^+ (0)^(*ξ*) and *ζ*_*u*_^− (0)^(*ξ*) in (61) and (62), Respectively, for the Limit of ***ξ*** → ∞

Although it is very difficult to analytically solve the characteristic Eqs. (61) and (62) for *ζ*_*u*_^+ (0)^(*ξ*) and *ζ*_*u*_^− (0)^(*ξ*), respectively, their approximate behaviors for the limit of *ξ* → ∞ can be analytically obtained; they are helpful for preparing figure-of-merit functions used as simple functions fitted to numerically calculated data of *ζ*_*u*_^+ (0)^(*ξ*) and *ζ*_*u*_^− (0)^(*ξ*). From (A.6), we find that ${U}^{\hbox{'}}\left(a,z\right)/U\left(a,z\right)\sim -\sqrt{a}$ for *a* → ∞, where *U* ^'^(*a*, *z*) = ∂_*z*_ *U*(*a*, *z*). From (A.21), it is inferred that ${w}_1^{\hbox{'}}\left(a,z\right)/{w}_1\left(a,z\right)\sim \sqrt{a}$ for *a* → ∞ and *z* ≠ 0, where *w*_1_^'^(*a*, *z*) = ∂_*z*_ *w*_1_(*a*, *z*). In a very similar way, from (A.21), it is inferred that ${w}_2^{\hbox{'}}\left(a,z\right)/{w}_2\left(a,z\right)\sim \sqrt{a}$ for *a* → ∞ and *z* ≠ 0, where *w*_2_^'^(*a*, *z*) = ∂_*z*_ *w*_2_(*a*, *z*). Therefore, we see that the characteristic Eqs. (61) and (62) for *ξ* → ∞ are written in the form

B.1 $$ \frac{1}{\varepsilon_d^{1/2}}\sqrt{\frac{\zeta_u^{\pm (0)}\left(\xi \right)+{\lambda}_0^2{\beta}_d^2\xi }{4\pi {\varepsilon}_d^{1/2}}}=-\frac{1}{{\left|{\varepsilon}_m\right|}^{1/2}}\left(-\sqrt{\frac{\zeta_u^{\pm (0)}\left(\xi \right)+{\lambda}_0^2{\beta}_m^2\xi }{4\pi {\left|{\varepsilon}_m\right|}^{1/2}}}\right) $$

(double-sign corresponds), which can be transformed into

B.2 $$ {\zeta}_u^{\pm (0)}\left(\xi \right)=4{\pi}^2{\left({\varepsilon}_d^{1/2}+{\left|{\varepsilon}_m\right|}^{1/2}\right)}^{-1}{\left({\varepsilon}_d^{-3/2}-{\left|{\varepsilon}_m\right|}^{-3/2}\right)}^{-1}\xi . $$

Equation (B.2) indicates that *ζ*_*u*_^± (0)^(*ξ*) behaves as a straight line with a slope of 4*π* ^2^(*ε*_*d*_^1/2^ + |*ε*_*m*_|^1/2^)^− 1^(*ε*_*d*_^− 3/2^ − |*ε*_*m*_|^− 3/2^)^− 1^ for *ξ* → ∞.
